# Melatonin Alleviates Low-Temperature Stress *via* ABI5-Mediated Signals During Seed Germination in Rice (*Oryza sativa* L.)

**DOI:** 10.3389/fpls.2021.727596

**Published:** 2021-09-27

**Authors:** Ruiqing Li, Meng Jiang, Yue Song, Huali Zhang

**Affiliations:** ^1^College of Agronomy, Anhui Agricultural University, Hefei, China; ^2^State Key Laboratory of Rice Biology and Chinese National Center for Rice Improvement, China National Rice Research Institute, Hangzhou, China; ^3^National Key Laboratory of Rice Biology, Institute of Crop Sciences, Zhejiang University, Hangzhou, China

**Keywords:** rice, seed germination, low temperature, melatonin, ABI5

## Abstract

With increasing areas of direct sowing, low-temperature (LT) stress drastically affects global rice production. Exogenous applications of melatonin (MT) serve as one of the effective ways to improve seed germination under various stress conditions. In this study, we found that MT treatment greatly improved the LT stress-induced loss of germination percentage and the weak performance of seedlings under LT of constant 20°C (LT20). This was largely dependent on the activated antioxidant system and enhanced activities of storage substance utilization-associated enzymes. Moreover, we also detected that exogenous feeding of MT significantly increased the biosynthesis of gibberellin (GA) and endogenous MT but simultaneously inhibited the accumulation of abscisic acid (ABA) and hydrogen peroxide (H_2_O_2_) under LT20 stress. These results suggested that MT had antagonistic effects on ABA and H_2_O_2_. In addition, MT treatment also significantly enhanced the expression of *CATALYSE 2* (*OsCAT2*), which was directly regulated by ABA-INSENSITIVE 5 (OsABI5), a core module of ABA-stressed signals, and thus promoting the H_2_O_2_ scavenging to reach reactive oxygen species (ROS) homeostasis, which consequently increased GA biosynthesis. However, in *abi5* mutants, *OsCAT2* failed in response to LT20 stress irrespective of MT treatment, indicating that OsABI5 is essential for MT-mediated seed germination under LT20 stress. Collectively, we now demonstrated that MT showed a synergistic interaction with an ABI5-mediated signal to mediate seed germination, partially through the direct regulation of *OsCAT2*.

## Introduction

Seed germination is vital for the subsequent establishment of seedlings in higher plants (Bewley, 1997). Some favorable environmental conditions, including at least optimal temperature, water, and oxygen, are required for the initiation of seed germination (Gutterman, 2002). However, due to their sessile lifestyle, plants may encounter various abiotic stress conditions and are forced to delay seed germination during the sowing seasons (Baskin and Baskin, 1998; Ahmad et al., 2012). It is well known that temperature plays a crucial role in the determination of germination under field conditions (Batlla and Benech-Arnold, 2010), whereas extreme temperature (i.e., cold and heat) makes negative effects on the metabolic process and water absorption, and consequently leads to a huge loss of germination rates during seed germination (Lou et al., 2007). For example, the rate of germination was largely prohibited or reduced in wheat (Cheng et al., 2009) and *Triticum aestivum* (Yamamoto et al., 2008) under high-temperature conditions. At the same time, seeds failed to germinate at low temperatures (LTs) in melon (Edelstein and Kigel, 1990) and *Citrullus colocynthis* (El-Keblawy et al., 2019).

Due to its tropical and subtropic origins, *Oryza sativa* L. is very susceptible to LT during the developmental stages (Hussain et al., 2016), and seed germination would be greatly delayed when the temperature is less than 15°C in rice (Fujino et al., 2004). LT stress drastically threatens the rice output of over 15 Mha all over the world (Lou et al., 2007). Nowadays, the race between increasing areas of direct sowing and increasing unstable climate during rice seedling (Iwata et al., 2010; Hussain et al., 2016; Wang et al., 2018) emphasizes the urgency to solve the challenges of rice cultivation at LTs, especially in several temperate Asian countries.

Exogenous applications of melatonin (*N*-acetyl-5-methoxytryptamine, MT) have been determined as an effective way to improve seed germination under various stress conditions (Hernández et al., 2015; Xiao et al., 2019; Simlat et al., 2020). For example, exogenous MT enabled the promotion of seed germination under salinity (Zhang et al., 2014) and water stress (Zhang et al., 2013) in cucumber (*Cucumis sativus* L.) while high levels of germination under post-salt and toxic copper ion stress conditions were also observed in cotton (*Gossypium hirsutum* L.) (Chen et al., 2020) and in red cabbage (Posmyk et al., 2008), respectively. Moreover, MT treatments also protected seeds against heat stress in *Arabidopsis* (Hernández et al., 2015) and salt stress in *Stevia rebaudiana* Bertoni (Simlat et al., 2020). In addition, improvements of MT on seed germination have also been reported in maize under chilling stress (Cao et al., 2019).

Germination involves a series of phytohormones in abiotic stress signaling such as abscisic acid (ABA), gibberellin (GA), and MT (Ahmad et al., 2012; Kanwar et al., 2018). Notably, the cross talk of MT with other phytohormones and stress makers constitutes a very complex regulatory network (Kanwar et al., 2018). Among these, ABA and its downstream signal components, including ABSCISIC ACID-INSENTIVE3 (ABI3) and ABI5, have been established as the downstream signals of MT in the regulation of antioxidant response to adverse conditions (Kanwar et al., 2018). MT may also regulate seed germination by improving antioxidant systems and scavenging reactive oxygen species (ROS) (Posmyk et al., 2008; Zhang et al., 2013). Moreover, ABI5 has been revealed to regulate seed germination partially through the homeostasis of ROS in *Arabidopsis* (Bi et al., 2017) and in barely (Ishibashi et al., 2017). Recently, Lv et al. (2021) reported that high concentrations of MT suppressed seed germination through its interrelationships with ABA, GA, and auxin under normal conditions in *Arabidopsis*. These studies confirmed that MT was involved in regulating seed germination *via* ABA signals. However, the possible regulatory mechanism of MT on seed germination *via* the balance of ABA and ROS remains largely unknown at LT in rice. Therefore, we now focused on the balance between MT and ROS under LT stress during rice seed germination.

## Materials and Methods

### Plant Materials

To generate *abi5* mutants, the 1st exon of *OsABI5* (*Os01g0859300*) was selected as a target (Supplementary Figure 1). The single-guide RNAs (sgRNAs) were designed by searching UniProt for precise positions^1^, and the CRISPR-P program^2^ was used to minimize the off-target effect (Lei et al., 2014). Rice calli was induced from mature seeds of the cultivar *O. sativa* L. *japonica* and was transformed with the pH-*osabi5* vector by *Agrobacterium-*mediated transformation. The cultivar *O. sativa* L. *japonica* was used as the wild type (WT).

The working MT solution was prepared as follows. About 0.175 g MT (Sangon Biotech, Shanghai, China) was firstly dissolved in 3 ml of ethyl alcohol, and then the fully dissolved solution was added with distilled water (DW) up to 500 ml, which served as the stock solution of 1,500 μmol/L. When in use, the working solution was prepared by diluting the stock solution of 1,500 μmol/L with DW (v:v = 1:9) until the final concentration reaching 150 μmol/L. The concentration of 150 μmol/L was obtained according to previous reports (Li X. et al., 2017).

For experimental treatments, the sterilized seeds were evenly placed on a 9-cm petri dish and then supplemented with 10 ml of DW with and without 150 μmol/L MT. Subsequently, all seeds were incubated for 7 days (Li et al., 2019) in the artificial chambers (12 h PAR, 300 μmol photons m^–2^ s^–1^ light/12 h dark; relative humidity: 65–75%) with the following temperature conditions: (i) 30/30°C (as the control check, CK); (ii) 24/24°C (as LT at 24°C, LT24); (iii) 23/23°C (as LT at 23°C, LT23); (iv) 20/20°C [as LT at 20°C (LT20)]; (v) 16/16°C (as LT at 16°C, LT16); and (vi) 15/15°C (as LT at 15°C, LT15). About 60 seeds derived from the same mother plant were served as one biological replicate, and six biological repeats were employed per time for each treatment. Seed germination was observed and recorded daily within the investigated days, and the evaporated water was added by the weighing method.

### Determination of Seed Morphological Index

Germinated seeds were evaluated according to the conditions that (i) the seed coat was broken, (ii) the length of radicle over exceeded the seed length, and (iii) the plumule length at least reached half of the seed length. The germination number of seeds was recorded every day in the germination period. Germinability was counted on the 5th day, whereas the germination percentage was counted on the 7th day. Seedlings were selected from each treatment to measure moisture absorption, healthy seedling rates, radicle length, plumule length, and fresh weight on the 7th day. The germination index and vital index were calculated according to the method as mentioned in a previous study (Liu and Liu, 2018).

### Quantification of the Associated Physiological Attributes

To determine physical efficiency, seedlings on the 7th day were dried at 105°C for 15 min and 85°C for 24 h (Zhu et al., 2012), and the weights were recorded for calculation.

Respiratory rates were measured using the devices of LI-6400XT system (LI-COR Biosciences, Lincoln, NE, United States) (Wang et al., 2005).

Membrane permeability was determined using the conductometry method (Liu and Zhu, 2014). Briefly, the cutup pieces of 0.3-g plumules were fully immersed into DW through a sealed vacuum. After being placed at room temperature for 60 min, the conductance values of *S*_1_ were determined by using the conductometer (DDS-11C). The abovementioned samples were then transferred to a boiling water bath for 10 min, and the conductance values of *S*_2_ were detected after cooling to room temperature. The conductance values of DW (*S*_0_) were used as a blank control. Relative conductance (*L*) was calculated as follows: *L* = (*S*_1_ − *S*_0_)/(*S*_2_ − *S*_0_).

Contents of the soluble sugar were analyzed by using the anthrone colorimetric method described by Fairbairn (1953). Briefly, 0.3 g of plumules were immersed into the DW and then boiled for 30 min in a water bath to collect the extracts. After adding sulfuric acid and anthrone, the absorbances of the extracts at 620 nm were determined to calculate the contents by a standard curve.

Contents of the soluble protein were determined with the Coomassie brilliant blue g-250 (G-250) as previously described (Sedmak and Grossberg, 1977). In brief, 0.3 g of plumules were ground in 1 M of pre-cold phosphatic buffer solution (containing 0.1% PVP, 0.1 M ethylenediaminetetraacetic acid (EDTA), 1 M ascorbic acid, pH 7.8), and then centrifuged at 15,000 *g* for 30 min at 4°C. The supernatant was collected and then incubated with G-250 for 2 min. The absorbances at 595 nm were measured to calculate the protein contents by a standard curve.

To determine the free proline contents (Bates et al., 1973; Song et al., 2021), 0.3 g of plumules were homogenized in 3% aqueous sulfosalicylic acid, and the filtered homogenate was collected to incubate in a boiling water bath for 10 min. After cooling to room temperature, the filtrate was centrifuged at 5,000 *g* for 10 min. The supernatants were continuously reacted with 2.5% ninhydrin and glacial acetic acid at 100°C for 60 min. After terminated in an ice bath, the reaction was added with 4 ml toluene. The chromophore phase was collected to measure the absorbance at 520 nm. The proline concentration was determined by using a standard curve.

Phospholipid contents were determined by using the Phospholipid Assay Kit (ab234050, Colorimetric, Abcam, Shanghai, China) according to the instructions of the manufacturer.

Acid values were determined using the method of potassium hydroxide (KOH) titration (Szafrańska, 2015). Briefly, 0.3-g plumules were ground in liquid nitrogen and totally solvent into the mixture of ethanol-diethyl ether. The mixture was then added with a phenolphthalein-alcohol indicator and was subsequently titrated with 0.1 M KOH until the reddish color would not disappear in the time period of 30 s.

### Determination of Enzymic Activities

Acid phosphatase (ACP) activity was measured using the method previously described by Olde Venterink (2011). Briefly, 0.1 g of plumules were homogenized in an ice-cold buffer [50 mM acetic acid-sodium acetate, pH 5.8, 50 mM-mercaptoethanol, 12.5% (v/v) glycerol], and the homogenate was then centrifuged at 20,000 *g* for 10 min at 4°C. The supernatant was incubated with 5 mM para-nitrophenylphosphate (p-NPP) at 30°C for 30 min, and then the reaction was terminated with 0.3 M NaOH. The absorption at 405 nm was measured to determine the ACP activity with a standard curve.

To detect the activity of cytochrome oxidase (CCO), 0.1 g of samples were homogenized in an ice-cold buffer [50 mM HEPES-NaOH, pH 7.4, 2 mM MgCl_2_, 50 mM mercaptoethanol, 12.5% (v/v) glycerol], and the homogenate was then centrifuged at 20, 000 *g* for 10 min at 4°C. The supernatants were used for enzyme activity assays with the Cytochrome C Oxidase Assay Kit (ab239711, Abcam, Shanghai, China) according to the instructions of the manufacturer.

The activity of succinic dehydrogenase (SDH) was determined spectrophotometrically using 2,6-dichlorophenolindophenol (DCPIP) according to the method mentioned in a previous study (Robinson and Lemire, 1995). Briefly, 0.1 g of plumules were homogenized in an ice-cold buffer [50 mM phosphate buffer, pH 7.4, 0.4 M sucrose, 10 mM EDTA, 50 mM-mercaptoethanol, 12.5% (v/v) glycerol], and the homogenate was then centrifuged at 20,000 *g* for 10 min at 4°C. The supernatant was incubated with the reaction medium (containing 20 mM phosphate buffer, pH 7.2, 0.1% Triton X-100, 4 mM sodium azide, 50 mM DCPIP) and 10 mM succinate at 37°C for 10 min. The reduction of DCPIP at 600 nm was used to calculate SDH activity by using the molar absorption coefficient of reduced DCPIP (21.0 mM^–1^ cm^–1^).

The activity of α-amylase was determined according to the method described in a previous study (Ishimoto et al., 2015). Briefly, 0.1 g of plumules were ground in a mortar with 20 mM sodium phosphate buffer (pH 6.7), and then centrifuged at 15,000 *g* for 10 min at 4°C. The supernatants were incubated with the substrate solution (1% potato starch, 20 mM NaCl, 0.1 mM CaCl_2_) for 15 min at 30°C. The reaction was then terminated by the addition of 3,5-dinitrosalicylic acid reagent in a boiling water bath for 10 min. The absorbance was measured at 546 nm, and α-amylase activity was expressed as micromoles of maltose liberated per minute.

### Determination of Antioxidant Activity

The activities of antioxidant enzymes were performed using the methods mentioned in a few previous studies (Zhou et al., 2014; Jiang et al., 2021) with little modifications. Briefly, the homogenized powder with liquid nitrogen from 0.3 g plumules was suspended with 50 mM pre-cold phosphate buffer (containing 0.2 mM EDTA, 2% polyvinylpyrrolidone (W/V), pH 7.8), and then immediately centrifuged at 4°C for 10 min (12,000 *g*). Subsequently, the supernatants were collected and used for the determination of the activity assays of superoxide dismutase (SOD, EC 1.15.1.1), catalase (CAT, EC 1.11.1.6), and peroxidase (POD, EC 1.11.1.7).

### Quantification of Malondialdehyde and Hydrogen Peroxide

Malondialdehyde (MDA) content was determined according to the methods proposed in a previous study (Heath and Packer, 1968) with 0.1 g of plumules. Quantification of hydrogen peroxide (H_2_O_2_) concentration was derived according to the methods of Velikova et al. (2000) with 0.1 g of plumules.

### Measurement of Endogenous Melatonin, Abscisic Acid, and Gibberellin Contents

Melatonin was extracted with 0.1 g of plumules according to the method mentioned in a previous study (Pape and Lüning, 2006) and was determined using an ELISA kit (Engvall and Perlmann, 1971) (Shanghai Enzyme Biotechnology Co., Ltd., Shanghai, China) following the instructions of the company.

The quantification of both ABA and GA was derived according to the methods mentioned in a previous study (Jahan et al., 2021) with little modifications. For endogenous ABA, 0.1 g of plumules were ground in liquid nitrogen and mixed with pre-cold extraction solution (methanol/water/formic, 16:3:1, v/v/v). After the centrifugation at 12,000 *g* for 15 min at 4°C, the supernatants were resuspended with 80% methanol and then filtered through a Sep-Pak C18 cartridge (Waters, Milford, MA, United States). The filtrates were collected for the measurement of the relative contents of ABA using the ELISA kit (Qingdao Sci-Tech Innovation Quality Testing Co., Ltd., Qingdao, China) according to the protocols of the manufacturer.

For endogenous GA, 0.1 g of plumules were ground in liquid nitrogen and incubated with pre-cold extraction solution (containing 80% methanol, 5% formic, 1 mM butylated hydroxytoluene) for 4 h at 4°C. After the centrifugation at 3,500 *g* for 15 min at 4°C, the supernatant was resuspended with 80% methanol and then filtered through a Sep-Pak C18 cartridge (Waters, Milford, MA, United States). Subsequently, all the filtrates were used for the quantification of the relative GA contents using the ELISA kit (Qingdao Sci-Tech Innovation Quality Testing Co., Ltd., Qingdao, China).

### Quantitative Real-Time PCR

Total RNA was extracted with 0.1 g of plumules by using the Qiagen Spin Plant RNA Mini kit (Qiagen, Hilden, Germany). Quantitative real-time PCR (qRT-PCR) analysis was performed according to the method previously proposed by Li R.Q. et al. (2017) and Li et al. (2021). Relative gene expression was calculated in relative to the rice *UBQ5 gene* (Jain et al., 2006) using the 2^–ΔΔCt^ method (Livak and Schmittgen, 2001). Gene-specific primers for qRT-PCR were listed in Supplementary Table 1.

### Electrophoretic Mobility Shift Assay

The full-length coding sequence of *OsABI5* was cloned into the pCZN1 vector to construct the combined OsABI5–pCZN1 vector, which was then transferred to *Escherichia coli* (Arctic-Express, Hilliard, OH, United States) for the production of recombinant protein. Glutathione–Sepharose 4B beads (GE Healthcare, Buckinghamshire, United Kingdom) were employed to collect the purified protein from the lysate cells. In parallel, 25-bp DNA fragments from the *OsCAT2* promoter [harboring the ABA response element (ABRE) motif, − 505th to − 481th distance to transcription start site (TSS)] were biotin-labeled at the 3′-end by using the electrophoretic mobility shift assay (EMSA) Probe Biotin Labeling kit (Promega, Madison, WI, United States). Unlabeled probes were subjected to cold competition experiments. The cold probe concentrations were 100 ×. EMSA was performed using the Light Shift Chemiluminescent EMSA kit (Promega, Madison, WI, United States) according to the instructions of the manufacturer. The EBNA protein was used as a negative control. The biotin signals were imaged using the ChemiDoc MP Imaging System (Bio-Rad Laboratories, Inc., Hercules, CA, United States). EMSAs were repeated three times, and the representative results were shown.

### Analysis of Genes for Their Expression

Genes were identified through a homolog search of the Rice Annotation Project (RAP) database^3^ : ABA-insensitive 5 (*OsABI5*, Os01g0859300); tryptophan decarboxylase (*OsTDC*, Os08g0140300); tryptamine 5-hydroxylase (*OsT5H*, Os12g026800); *O*-methyltransferase (*OsASMT*, Os09g0344500); serotonin *N*-acetyltransferase (*OsSNAT*, Os05g0481000); abscisic aldehyde oxidase (*OsAAO*, Os07g0281700); cytochrome P450 99A3 (*OsCYP99A3*, Os04g0178400); 9-*cis-*epoxycarotenoid dioxygenase 1 (*OsNCED1*, Os02g0704000); *OsNCED2* (Os12g0435200); *OsGA1* (Os05g0333200); *OsGA2ox1* (GA 2-oxidase1, Os05g0158600); *OsGA2ox2* (Os01g0332200); *OsGA2ox3* (Os01g0757200); *OsGA2ox4* (Os05g0514600); and catalase-2 (*OsCAT2*, Os02g0115700). These genes were subjected to qRT-PCR analysis by using gene-specific primers (Supplementary Table 1).

### Statistical Analysis

Values were expressed as means ± SEs with six biological replicates. Comparisons of data from different groups were analyzed using the ANOVA test followed by the Tukey’s multiple comparison test with *p* < 0.05.

## Results

### Effects of Melatonin Treatment on the Germination Rate Under Different Low-Temperature Stress Conditions

**FIGURE 1:**
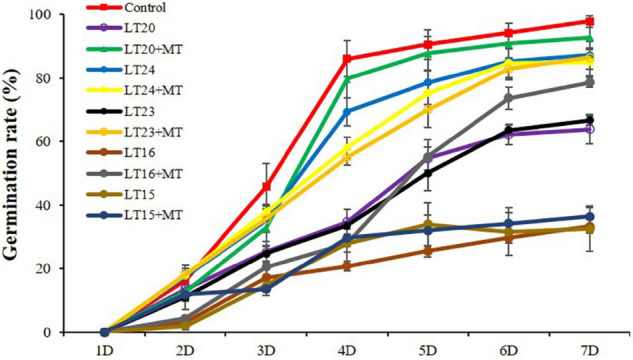
Germination rates of rice seed germination during different low-temperature (LT) stress conditions in the presence or absence of melatonin (MT) treatment. Exogenous applications of MT (150 mM) were employed to provide treatment for rice seeds under different LT stress conditions for 7 days. Control: constant 30°C; LT15: constant 15°C; LT20: constant 20°C; LT24: constant 24°C.

To determine the effects of MT treatment on the seed germination rates under LT stress, the seeds of WT were treated with MT under different LTs (depicted as LT15, LT16, LT20, LT23, and LT24). Compared with the control, the germination rates were greatly inhibited under all LT conditions (Figure 1). However, no significant difference of the germination rates was detected between feeding with and without MT under LT15 and LT24 conditions (Figure 1). In addition, more accumulation of H_2_O_2_ and MDA was detected at LT15 (Supplementary Figure 2), making severe effects on the seed germination. Conversely, the feeding of MT greatly improved the germination rates under LT16 and LT23 stress, reaching 2.36 fold and 1.30 fold, respectively, in relative to non-treatment groups on the 7th day (Figure 1). Especially, under LT20 conditions, the exogenous MT greatly increased the germination rates within the investigated 3–7 days (Figure 1). Thus, the middle one, LT20, within the LT of 16–23°C was selected, and the germinated seeds on the 7th day under LT20 stress were employed for further investigation.

### Effects of Melatonin Treatment on Seed Germination Under Low-Temperature Stress

**FIGURE 2:**
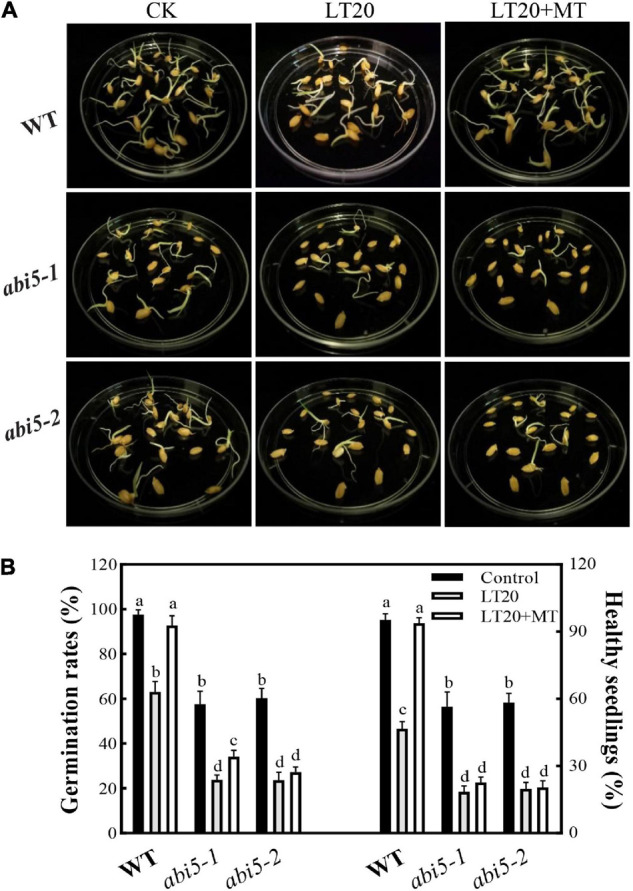
Effects of exogenous MT treatment on the seed germination performance under LT stress. **(A)** Phenotypes, **(B)** germination percentage, and healthy seedling rates under LT stress (constant 20°C for 7 days) without (LT20) or with (LT20 + MT) MT treatment. Different letters denote significant variations between the treatments, and the average values were measured by Tukey’s honestly significant difference (HSD) test at *p* < 0.05. Data were represented as mean ± SE of six biological replicates.

As shown in Figure 2A, after LT20 treatment, seed germination was suppressed in WT, *abi5-1*, and *abi5-2*. Nonetheless, the feeding of MT greatly rescued the phenotypical performances of WT, but no significant difference was observed in *abi5-1* and *abi5-2* (Figure 2A).

To further explore the effects of *OsABI5* mutation on the MT alleviation under LT20 stress, the germination-associated index was studied (Figure 2B, Supplementary Figures 3A,B, and Supplementary Table 2). Compared with the control, all the investigated indices in WT were significantly reduced after LT20 treatment while the MT addition greatly relieved the damages of LT20 to seed germinability, germination percentage, and germination index (Figure 2B and Supplementary Figures 3A,B), as well as seedling performance, including vital index, moisture absorption, and healthy seedling rates (Figure 2B, Supplementary Figures 3C,D, and Supplementary Table 2). However, in *abi5-1* and *abi5-2*, the investigated index under LT20 stress were significantly lower than the control. The germination percentage (Figure 2B), germinability, and germination index of seed (Supplementary Figures 3A,B), as well as healthy seedling rates (Figure 2B), vital index, and moisture absorption (Supplementary Figures 3C,D), were rarely or never recovered in the conditions of MT incubation in *abi5-1* and *abi5-2*. These results suggested that MT treatment relieved LT20-induced damages to the performance of seed germination, and that *ABI5* mutation greatly affected the alleviation effects of MT on LT20 stress.

### Effects of Melatonin Treatment on the Physiological Attributes Associated With Seed Germination

**FIGURE 3:**
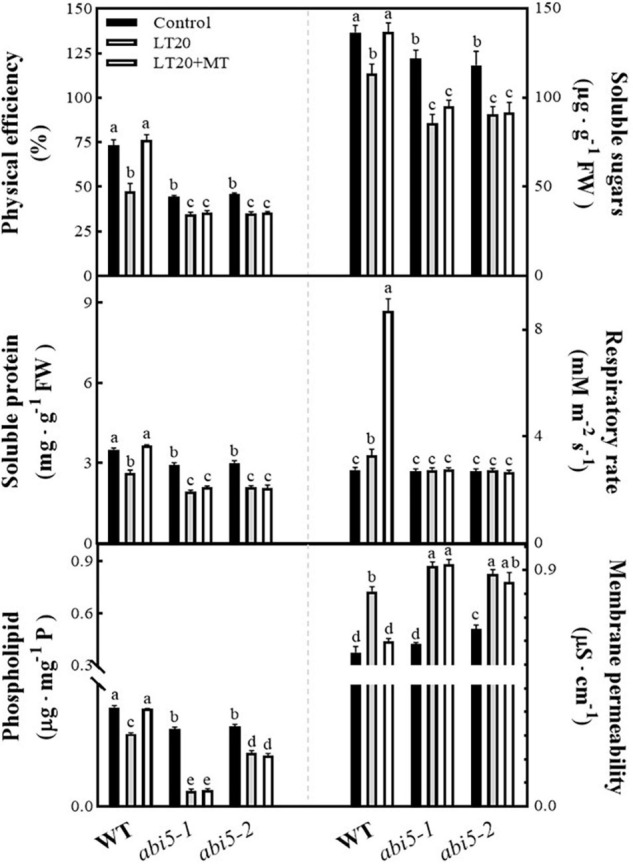
Effects of exogenous MT treatment on seed germination-associated physiological attributes under LT stress. Physical efficiency, soluble sugar contents, soluble protein contents, phospholipid contents, respiratory rate, and membrane permeability under LT stress (constant 20°C for 7 days) without (LT20) or with (LT20 + MT) MT treatment. Different letters denote significant variations between the treatments, and the average values were measured by Tukey’s HSD test at *p* < 0.05. Data were represented as mean ± SE of six biological replicates.

Because seed germination requires the nutritive materials (i.e., soluble sugar and soluble protein) derived from the enzymatic decomposition of the storage materials as well as the energy provided by respiration, thus the physiological attributes associated with germination, including soluble sugar, soluble protein, physical efficiency, and respiratory rates, were studied to explore the physiological effects of MT on LT20 stress (Figure 3 and Supplementary Table 3). Compared with CK, the physical efficiency and the soluble sugar contents, soluble protein contents, and phospholipid contents were significantly reduced under LT20 stress in WT, whereas the MT feeding greatly mitigated the decreased trends (Figure 3). In addition, the respiratory rates were also significantly enhanced after LT20 treatment but reached much higher in the presence of MT feeding (Figure 3). In contrast, the acid value (Supplementary Figure 4) and membrane permeability (Figure 3) were significantly increased under LT20 treatment but were reduced to the control levels in WT once feeding with MT. Consistent with Figure 2, these results suggested that exogenous MT alleviated the adverse effects of LT20 stress on seed germination.

However, in *abi5-1* and *abi5-2*, no significant difference was detected in the investigated physiological attributes under LT20 stress irrespective of MT feedings (Figure 3 and Supplementary Figure 4). Besides, except for the respiratory rates (Figure 3), the four positive (Figure 3) and the two negative (Figure 3 and Supplementary Figure 4) correlated attributes for seed germination showed significant differences in relative to control under LT20 stress. These results suggested that *OsABI5* mutation greatly repressed the alleviating effects of MT on LT20 stress during seed germination.

### Effects of Melatonin Treatment on the Enzymic Activities Associated With Germination

**FIGURE 4:**
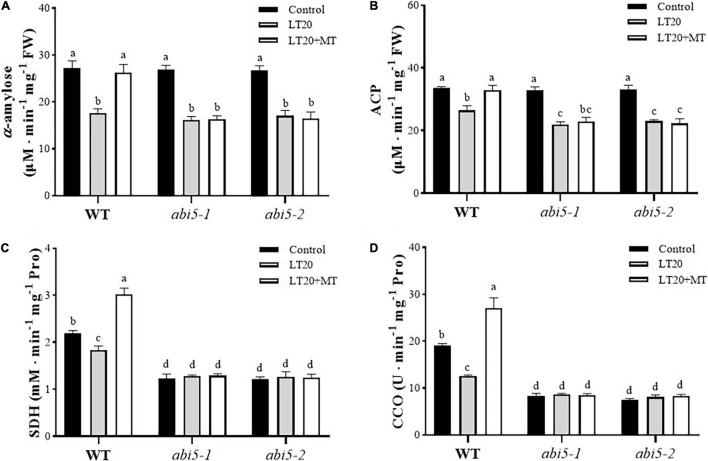
Effects of exogenous MT treatment on seed germination associated with enzymes under LT stress. Activities of **(A)** α-amylase, **(B)** acid phosphatase (ACP), **(C)** succinic dehydrogenase (SDH), and **(D)** cytochrome oxidase (CCO) under LT stress (constant 20°C for 7 days) without (LT20) or with MT (LT20 + MT) treatment. Different letters denote significant variations between the treatments, and the average values were measured by Tukey’s HSD test at *p* < 0.05. Data were represented as mean ± SE of six biological replicates.

Because enzyme catalysis was required for the degradation of storage substances and respiration reaction during seed germination (Bewley, 1997), thus we next detected the effects of MT on the activities of partial enzymes involved in seed germination, including α-amylase and ACP used for storage metabolism as well as SDH and CCO for respiration (Figure 4 and Supplementary Table 4). After LT20 treatment, the activities of α-amylase, ACP, SDH, and CCO were significantly decreased in WT, whereas the incubation of MT greatly enhanced the activities of these four enzymes (Figures 4A–D).

However, in *abi5-1* and *abi5-2*, the activities of α-amylase and ACP were significantly decreased after LT20 stress, but no significant difference was detected between feeding with and without MT under LT20 (Figures 4A,B). Interestingly, no significant difference in the activities of SDH and CCO was found between CK and LT20 stress in *abi5-1* and *abi5-2* mutants, even in the conditions of MT feedings (Figures 4C,D). Thus, these results suggested that *OsABI5* mutation severely affected the alleviation effects of MT on LT20 stress to inhibit enzyme catalysis, which is required for the utilization of storage substances during seed germination.

### Effects of Melatonin Treatment on Reactive Oxygen Species Accumulation and Antioxidant Activity

**FIGURE 5:**
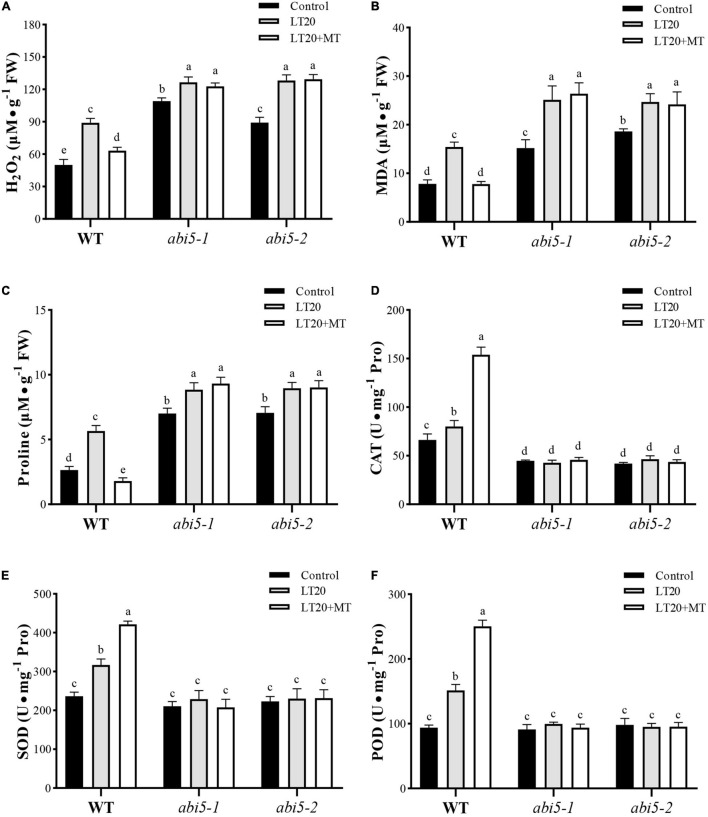
Effects of exogenous MT treatment on an antioxidant system under LT. **(A)** Hydrogen peroxide (H_2_O_2_) content, **(B)** malondialdehyde (MDA) content, **(C)** proline (Pro) content, **(D)** catalase (CAT) activity, **(E)** superoxide dismutase (SOD) activity, and **(F)** peroxidase (POD) activity under LT stress (constant 20°C for 7 days) without (LT20) or with (LT20 + MT) MT treatment in wild type (WT) and *abi5* mutants. Different letters denote significant variations between the treatments, and the average values were measured by Tukey’s HSD test at *p* < 0.05. Data were represented as mean ± SE of six biological replicates.

Low-temperature stress leads to the accelerated production of harmful metabolites and simultaneously activates the enzymatic antioxidant system to prevent cells from damage (Fujino et al., 2004; Hussain et al., 2016). Thus, we further detected the accumulation of MDA, H_2_O_2_, and free proline, as well as an antioxidant system under LT20 stress. As shown in Figures 5A,B, under LT20 stress, the concentration of MDA and H_2_O_2_ was greatly increased in all investigated samples, whereas MDA and H_2_O_2_ accumulated in *abi5* mutants were more than those accumulated in WT. Nonetheless, the incubation of MT with seeds under LT20 significantly suppressed the production of MDA and H_2_O_2_ in WT but failed to inhibit the accelerated accumulation of harmful metabolites in *abi5-1* and *abi5-2* (Figures 5A,B). In addition, in WT, the contents of free proline under LT20 stress were significantly increased in relative to CK but were restored to CK levels after MT treatment (Figure 5C). Similarly, in *abi5-1* and *abi5-2*, free proline detected under LT20 was more than that detected under CK, but MT treatment made no significant changes, indicating that MT failed to relieve LT stress in *abi5* mutants (Figure 5C).

Correspondingly, the activities of CAT, SOD, and POD showed antagonistic correlations with the accumulation of MDA and H_2_O_2_ under LT20 stress (Figure 5 and Supplementary Table 5). The activities of CAT, SOD, and POD in WT were significantly enhanced under LT20 as compared to CK, especially in the conditions of MT treatment (Figures 5D–F). However, in *abi5-1* and *abi5-2*, no significant difference was detected under LT20 stress compared with CK, irrespective of MT treatment, suggesting that *OsABI5* mutation suppressed the activities of an antioxidant system (Figures 5D–F). These results indicated that *OsABI5* mutation has negative effects on the MT functions and enables the promotion of LT20-induced production of harmful substances during seed germination.

### Effects of Exogenous Melatonin Treatment on the Biosynthesis of Endogenous Melatonin

**FIGURE 6:**
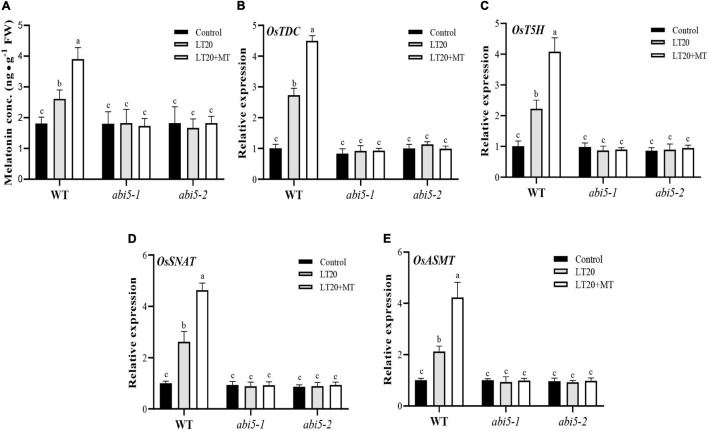
Effects of exogenous MT treatment on the biosynthesis of endogenous MT under LT. **(A)** Endogenous MT content and **(B–E)** transcript abundance of MT biosynthesis genes (*TDC*, *T5H*, *SNAT*, and *ASMT*) under LT stress (constant 20°C for 7 days) without (LT20) or with (LT20 + MT) MT treatment in WT and *abi5* mutants. Different letters denote significant variations between the treatments, and the average values were measured by Tukey’s HSD test at *p* < 0.05. Data were represented as mean ± SE of six biological replicates.

To explore the effects of *OsABI5* mutation on the biosynthesis of endogenous MT, the contents of endogenous MT and the expression levels of the genes involved in MT biosynthesis were further detected (Figure 6 and Supplementary Table 6). Under LT20 stress, the contents of endogenous MT in WT were increased by 105.6% and 55.6% in the presence and absence of MT, respectively, compared to CK (Figure 6A). However, in *abi5-1* and *abi5-2*, compared with CK, no significant difference in the contents of endogenous MT was detected under LT20 stress irrespective of MT treatment, suggesting that *OsABI5* mutation negatively affected the biosynthesis of endogenous MT (Figure 6A).

Moreover, the expression levels of *OsTDC*, *OsT5H*, *OsSNAT*, and *OsASMT* in WT were also increased by 2–2.9 folds under LT20 stress but significantly enhanced up to fourfold to 4.8 fold once MT feeding (Figures 6B–E). However, in *abi5-1* and *abi5-2*, there were no significant differences in transcriptional abundance presented under LT20 stress with or without MT (Figures 6B–E). All these results indicated that *OsABI5* mutation affects the expression of genes for the biosynthesis of endogenous MT under LT20 stress even in the conditions of exogenous MT treatment.

### Effects of Exogenous Melatonin Treatment on the Biosynthesis of Endogenous Abscisic Acid

**FIGURE 7:**
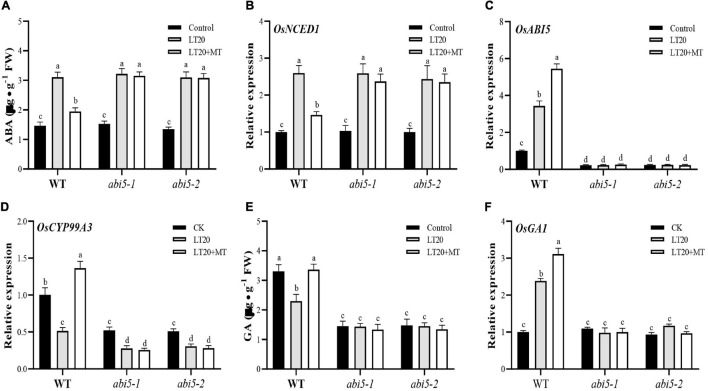
Effects of exogenous MT treatment on the endogenous abscisic acid (ABA) and gibberellin (GA) biosynthesis under LT. **(A)** Endogenous ABA content, **(B)** transcript abundance of ABA biosynthesis gene (*OsNCED1*), **(C)** ABA signaling gene [ABA INSENSITIVE 5 (*OsABI5*)], **(D)** ABA of catabolic gene (*OsCYP99A3*), **(E)** endogenous GA content, and **(F)** transcript abundance of GA signaling genes (*OsGA1*) under LT stress (constant 20°C for 7 days) without (LT20) or with (LT20 + MT) MT treatment in WT and the *abi5* mutants. Different letters denote significant variations between the treatments, and the average values were measured by Tukey’s HSD test at *p* < 0.05. Data were represented as mean ± SE of six biological replicates.

Seed germination requires the cooperation of different phytohormones, such as MT, ABA, and GA under various stress conditions (Ahmad et al., 2012), and it is well known that ABA serves as a positive regulator of ABI5 signals during seed germination (Skubacz et al., 2016; Bi et al., 2017). Thus, the contents of endogenous ABA and GA as well as the expression levels of genes involved in ABA and GA metabolism were further detected in the conditions with or without exogenous MT under LT20 stress (Figure 7 and Supplementary Tables 7, 8).

Under LT20 stress, the contents of ABA, and the expression levels of genes for ABA biosynthesis were significantly upregulated in WT, *abi5-1*, and *abi5-2* (Figures 7A,B and Supplementary Figures 5A,B). By contrast, under LT20 stress, the treatment of MT significantly decreased the ABA concentration and the expression of *OsNCED1*, *OsNCED2*, and *OsAAO* in WT while *abi5-1* and *abi5-2* remained to be in the same level as the single LT20 treatment irrespective of MT feedings (Figures 7A,B and Supplementary Figures 5A,B).

However, as a catabolic gene for ABA, *OsCYP99A3* showed contrast expression with the genes of ABA synthesis (Figure 7). LT20 treatment enhanced expression of *OsABI5* and reduced expression of *OsCYP99A3*, but the feeding of MT significantly induced the transcriptional levels of *OsABI5* and promoted the expression of *OsCYP99A3* (Figures 7C,D). Nonetheless, in *abi5-1* and *abi5-2*, no significant difference of transcriptional abundance of *OsABI5* was detected between LT20 stress and the control, even under MT treatment (Figure 7C). Moreover, in *abi5-1* and *abi5-2*, although the expression of *OsCYB99A3* was downregulated after LT20 treatment, no significant difference of the *OsCYB99A3* expression was detected under LT20 stress despite MT treatment (Figure 7D). All these results suggested that MT had antagonism effects on ABA under LT20 stress, which was weakened or failed due to *OsABI5* mutation.

### Effects of Exogenous Melatonin Treatment on the Biosynthesis of Endogenous Gibberellin

Compared with CK, GA contents under LT20 stress were significantly decreased by 35.3% in WT, whereas the feeding of MT significantly increased the GA contents to CK levels (Figure 7E). However, no change of GA concentration was detected under LT20 stress in *abi5-1* and *abi5-2*, even in the presence of MT (Figure 7E), suggesting that *OsABI5* mutation negatively affected the effects of MT in promoting GA synthesis.

Furthermore, the expression levels of *OsGA1* (Figure 7F) and the genes involved in GA catabolism (Supplementary Figures 6A–D) under LT20 stress were significantly enhanced in WT, but MT treatment led to a higher expression of *OsGA1* and decreased expression of *OsGA2ox1, OsGA2ox2, OsGA2ox3*, and *OsGA2ox4* (Figure 7F and Supplementary Figures 6A–D). Nonetheless, in *abi5-1* and *abi5-2*, no significant difference of transcriptional abundance of *OsGA1*, *OsGA2ox1*, *OsGA2ox2*, *OsGA2ox3*, and *OsGA2ox4* was detected under LT20 stress, irrespective of MT treatment (Figure 7F and Supplementary Figures 6A–D). These results suggested that MT had a synergistic interaction on GA biosynthesis, which was inhibited in *OsABI5* mutants.

### Regulation of OsABI5 on *OsCAT2* Expression

**FIGURE 8:**
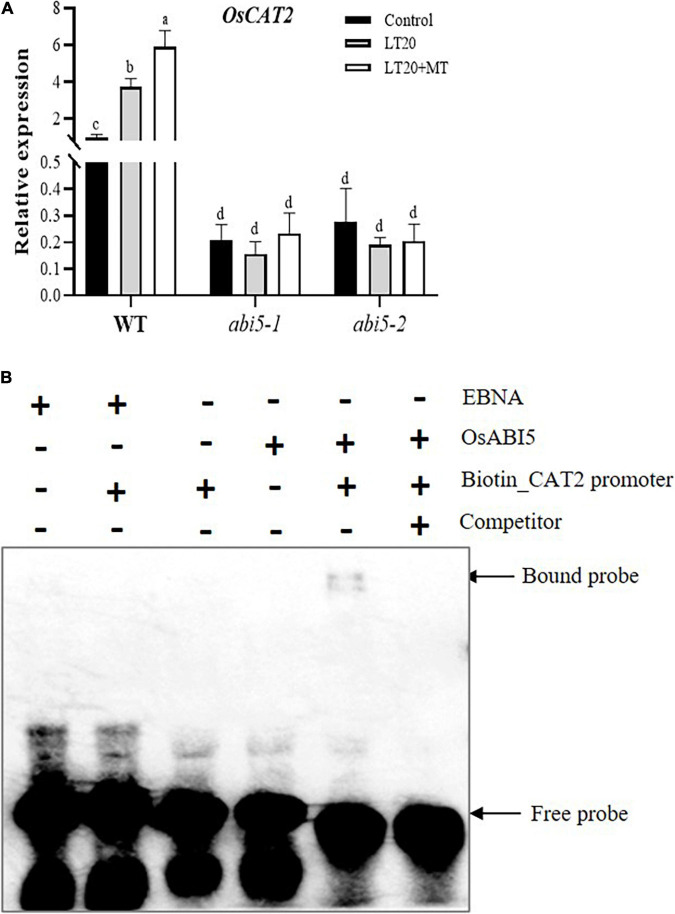
Expression of *CATALYSE 2* (*OsCAT2*) under LT stress treated with exogenous MT and electrophoretic mobility shift assay (EMSA) assays for a direct interaction between OsABI5 and the *OsCAT2* promoter. **(A)** Transcript abundance of *OsCAT2* under LT stress (constant 20°C for 7 days) without (LT20) or with MT (LT20 + MT) treatment in WT and *abi5-1* and *abi5-2* mutants. **(B)** An EMSA validated the interaction of OsABI5 with the promoters of *OsCAT2.* The purified OsABI5 protein obtained using prokaryotic expression was used for the *in vitro* assay, and the EBNA protein was used as a negative control. Unlabeled probes (competitor) were subjected to cold competition experiments (100×). Different letters denote significant variations between the treatments, and the average values were measured by Tukey’s HSD test at *p* < 0.05. Data were represented as mean ± SE of six biological replicates. “+” and “–” indicate the presence or absence, respectively, of proteins and probes in the loading mixture. About 4% of the polyacrylamide gel was used here.

The expression of *OsCAT2*, one-gene encoding catalysis, is closely associated with seed germination and dormancy (Bailly et al., 2008; Ishibashi et al., 2017). The expression of *OsCAT2* in WT was induced by LT treatment, whereas higher transcription abundance was detected once feeding with MT (Figure 8A). However, in *abi5-1* and *abi5-2*, no significant difference of *OsCAT2* expression was observed under LT20 stress with or without MT (Figure 8A). EMSA assay demonstrated that OsABI5 was directly bound to the promoter areas of *OsCAT2* (Figure 8B). The results suggested that the regulation of OsABI5 on the expression of *OsCAT2* was affected by MT under LT20 stress.

## Discussion

### Melatonin Alleviates Low-Temperature Stress During Seed Germination

Due to its origin from tropical and subtropical areas, rice is susceptible to temperature, especially for LT, which would greatly limit growth and yield (Hussain et al., 2016; Li and Yang, 2020). The optimal temperature for rice s germination is in the range of 25–35°C as the temperature of less than 15°C would greatly inhibit the germination rates (Fujino et al., 2004). Li and Yang (2020) found that the seed germination of two rice genotypes was greatly inhibited at an LT of 15°C. Currently, we also found that the germination rates of rice seeds were greatly decreased under different LT stress conditions, especially in the temperature below 20°C (Figure 1). For example, under LT15 stress, the germination of seeds was severely suppressed, with the disrupted cell metabolism and accumulated harmful substances (Figure 1 and Supplementary Figure 2). However, the feeding of MT did not greatly improve the germination rates at both 15°C and 24°C (Figure 1). Because LT stress (15°C) would severely disrupt the activity of enzymes being essential for seed germination, and cause the accumulation of oxidative products, i.e., MDA and H_2_O_2_ (Supplementary Figure 2), and thus the feeding of MT also could not restore the germination rates. Meanwhile, the temperature of 24°C was very close to the optimal temperature for rice seed germination, thus little effects were shown on the seed germination (Figure 1). Nevertheless, the germination rates were greatly inhibited under the LT of 16°C and 23°C, whereas they were greatly restored after feeding with exogenous MT, reaching 2.36 and 1.30 folds in relative to non-treatment groups (Figure 1). In addition, under LT20 stress, the middle temperature in the range of 16–23°C, the seed germination was also greatly improved after feeding with exogenous MT (Figures 1, 2). Therefore, MT can alleviate LT stress in the range of 16–23°C, and shows potential application values in production.

Seed dormancy or delayed germination is an adaptative response to adverse environments, and in turn, germination will be triggered when the conditions are favorable for seedling recruitments under natural environments (Clauss and Venable, 2000; Gutterman, 2002). This was greatly related to the counteraction of various bioactive phytohormones, such as GA and ABA (Miransari and Smith, 2014). Therefore, it was feasible to improve seed germination by using phytohormones, i.e., MT, in agricultural productions. Indeed, MT has been widely and effectively applied to alleviate the stress-induced delays of seed germination in various plants (Posmyk et al., 2008; Zhang et al., 2013, 2014; Hernández et al., 2015; Chen et al., 2020; Simlat et al., 2020). In this research, we found that the applications of exogenous MT significantly improved the germination rates (Figures 1, 2A,B and Supplementary Figures 3A,B) and seedling performance (Supplementary Figures 3C,D), which were largely related to the recovery of water absorption (Supplementary Figure 3D) and membrane permeability (Figure 3). However, under LT20 stress, the metabolism of storage substances and respiratory rate were also inhibited (Figure 3), but the acid value (Supplementary Figure 4) and harmful substances (Figures 5A,B) were greatly accumulated. On the contrary, the MT applications efficiently enhanced the supplies of nutrients (Figure 3), such as soluble sugars and proteins, as well as sufficient water (Supplementary Figure 3D) for seed germination under LT20 stress. Therefore, these results suggested that MT alleviated the LT -stress-induced inhibition of seed germination.

In addition, despite the support of moderate ROS to seed germination, over the accumulation of ROS and MDA would prevent or delay germination, especially under adverse conditions (Bailly et al., 2008; Wojtyla et al., 2016). Here, LT stress promoted the concentrations of H_2_O_2_ and MDA during germination while the treatment of MT greatly inhibited the production of LT stress-induced ROS (Figures 5A–C), which in turn enabled the promotion of germination rates (Figure 2B and Supplementary Figures 3A,B). Thus, the effects of MT on the LT stress germination were dependent on the effective functioning of an antioxidant system. Moreover, the interaction between H_2_O_2_ and ABA was involved in the regulation of seed germination and dormancy in barley (Ishibashi et al., 2017). In this research, the concentration of ABA showed consistent changes with the accumulation of H_2_O_2_ under LT stress (Figures 5A, 7A). In addition, the *OsCAT2* expression, directly regulated by OsABI5, was responsible for the CAT activity (Figure 8). However, the *OsCAT2* expression was even higher in the conditions of upregulated *OsABI5* under MT treatment (Figures 7E, 8). Interestingly, Ishibashi et al. (2017) demonstrated that ABA-induced HvABI5 enabled the promotion of CAT2, a H_2_O_2_ scavenging enzyme, to suppress the ROS signals for GA biosynthesis in dormant seeds. This seemed to be consistent with our results. Moreover, Mittler and Blumwald (2015) proposed that stress-induced ABA could synergistically enhance the ROS produced by NADPH oxidase in guard cells, which led to the accumulation of ABA in the positive feedback loop model. In addition, the regulation of OsABI5 on seed germination may be involved in other regulatory mechanisms, such as in the recently reported OsABI5/OsKEAP1 system (Liu et al., 2021). ABA contents between dormant seeds and LT-stressed seeds showed obvious differences, which might be served as other reasonable attributes.

All in all, LT stress greatly inhibited the seed germination while MT treatment could alleviate LT-induced damages to seeds *via* systemic acquired acclimation, i.e., enhanced antioxidant activity.

### Melatonin Alleviates Different Low-Temperature Stress Conditions *via* Cross Talk With Abscisic Acid During Seed Germination

The regulation of MT on abiotic-stressed seed germination at least involves two ways, by cross talk with other plant hormones (Zhang et al., 2014; Cao et al., 2019; Xiao et al., 2019; Chen et al., 2021) and by small molecular signals (e.g., ROS). For example, MT is involved in the regulation of ABA and GA to promote seed germination under salt stress (Zhang et al., 2014; Chen et al., 2021). It is well known that GAs and ABA play vital roles in seed germination and ABA acts as a key molecule in dormancy (Finkelstein et al., 2008; Rodríguez et al., 2015; Shu et al., 2016) while GAs prefer to promote seed germination (Jacobsen et al., 2002; Tuttle et al., 2015). Such an antagonistic interaction of GA and ABA was consistent with our results. In this study, LT treatment led to the biosynthesis of ABA, which in turn enabled the inhibition of the GA, thus inhibiting seed germination (Figures 2, 7 and Supplementary Figure 3). This was also concluded from the gene expression levels for ABA. For example, as the catabolic gene for ABA, *OsCYP99A3* was induced in the presence of moderate ROS contents (i.e., H_2_O_2_) during seed germination while over-accumulated ROS would greatly repress its expression, which had eventually helped to accumulate more ABA to inhibit seed germination (Figures 2, 5, 7). However, the feeding of exogenous MT not only increased the expression of *OsCYP99A3* to promote the catabolism of ABA but also greatly rescued the biosynthesis of GA and MT, which was beneficial to seed germination (Figures 2, 6, 7). On the other hand, LT stress also induced the accumulation of harmful substances, especially H_2_O_2_ (Figure 5A), which eventually broke the redox homeostasis that was essential for seed germination. MT has been proved to be an effective antioxidant for scavenging the excess ROS (Galano et al., 2011). In this case, the treatment of MT indeed repressed the production of H_2_O_2_ under LT conditions (Figure 5A). All these results suggested that MT was involved in the regulation of seed germination under LT stress, possibly *via* its antagonism with ABA. Nonetheless, MT showed a synergetic correlation with ABA to regulate seed germination under non-stress conditions (Wei et al., 2018; Lv et al., 2021), which was largely due to the different applied doses of MT.

Apart from various hormones, more and more studies have demonstrated that small molecules also played a function in the regulation of seed germination (Lv et al., 2021). For example, ABA acting as the major stress phytohormone is involved in the response of the seed to adverse environments during germination (Skubacz et al., 2016) while ABI5, a key module of the core ABA signaling, mediated seed germination partially through the ROS homeostasis (Bi et al., 2017). Moreover, as an effective antioxidant, phytomelatonin was supposed to regulate seed germination through ABI5-mediated ROS signals. There were two reasons to explain this point. Firstly, MT treatment has antagonistic effects on the LT stress-induced accumulation of ABA while *abi5* abolished the effects of MT on the biosynthesis of ABA (Figures 7A,B and Supplementary Figure 5). Secondly, under LT stress, MT greatly enabled the promotion of the CAT activity (Figure 5D), and the expression of its encoded gene, *OsCAT2*, which was directly regulated by OsABI5 (Figure 8A). This was also concluded from the failure of *OsCAT2* in response to LT stress in *abi5* mutants, even in the presence of MT feedings (Figure 8A).

**FIGURE 9:**
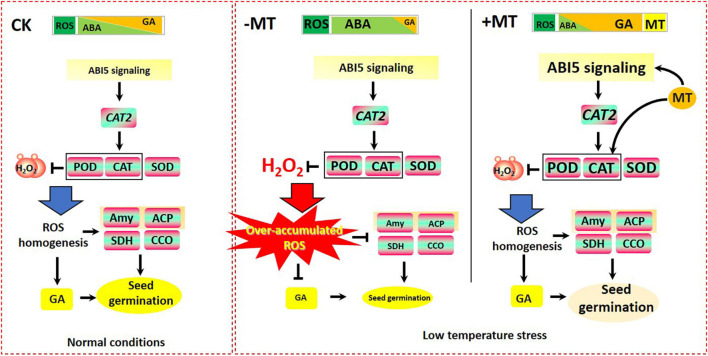
Models for explaining the alleviated effects of MT on LT-stressed seed germination in rice. During seed germination, LT stress caused the accumulation of reactive oxygen species (ROS) and ABA, which was helpful to maintain seed dormancy. However, the disruption of redox homeostasis caused by LT stress-induced ROS accumulation greatly suppressed the biosynthesis of GA and the efficient applications of storage materials (i.e., soluble sugar), to eventually prevent seed germination. However, MT treatment greatly activated OsABI5 to regulate *OsCAT2* and promoted the antioxidant systems (i.e., SOD, POD, and CAT) to scavenge ROS, which would consequently restore the steady states of ROS homeostasis in the germinating seeds; the homeostasis of ROS, in turn, enabled the promotion of the catabolism of ABA, which finally relieved the inhibitions for seed germination and simultaneously contributed to the biosynthesis of GA as well as the effective functioning of enzymes involved in seed germination (i.e., Amy, ACP, SDH, and CCO), and thereby promoting seed germination.

Therefore, in the context of seed germination, LT stress was found to result in the accumulation of ROS and ABA (Figures 5, 9), which was helpful to maintain seed dormancy, while the disruption of redox homeostasis caused by LT stress-induced ROS accumulation greatly suppressed the biosynthesis of GA (Figure 7E) and the efficient applications of storage materials (i.e., soluble sugar; Figures 3, 4 and Supplementary Figure 4), which eventually prevented seed germination (Figures 1, 2 and Supplementary Figure 3). However, MT treatment greatly activated OsABI5 to regulate *OsCAT2* and enabled the promotion of the antioxidant systems (i.e., SOD, POD, and CAT; Figures 5, 9) to scavenge ROS, which consequently restored the steady states of ROS homeostasis in the germinating seeds; the homeostasis of ROS, in turn, enabled the promotion of the catabolism of ABA (Figure 7), which finally relieved the inhibitions for seed germination and simultaneously contributed to the biosynthesis of GA (Figure 7) as well as the effective functioning of enzymes (Figures 4, 7), and thereby promoting seed germination (Figure 9).

## Conclusion

We now demonstrate that MT synergistically acts with an ABI5-mediated signal to regulate rice seed germination under LT stress, possibly through a direct regulator of *OsCAT2*.

## Data Availability Statement

The original contributions presented in the study are included in the article/Supplementary Material, further inquiries can be directed to the corresponding author/s.

## Ethics Statement

The authors declare that the experiments were performed in compliance with the current laws of China.

## Author Contributions

RL and MJ conceived the study. RL, MJ, and YS carried out the experiments and performed data analysis. RL, MJ, and HZ finished the first draft. RL and HZ finished the final version. All authors gave their consent for publication.

## Conflict of Interest

The authors declare that the research was conducted in the absence of any commercial or financial relationships that could be construed as a potential conflict of interest.

## Publisher’s Note

All claims expressed in this article are solely those of the authors and do not necessarily represent those of their affiliated organizations, or those of the publisher, the editors and the reviewers. Any product that may be evaluated in this article, or claim that may be made by its manufacturer, is not guaranteed or endorsed by the publisher.

## Footnotes

1
http://www.uniprot.org/

2
http://cbi.hzau.edu.cn/cgi-bin/CRISPR/

3
https://rapdb.dna.affrc.go.jp/

## Supplementary Material

Supplementary Figure 1 A schematic diagram of an ABA-INSENSITIVE 5 (*OsABI5*) and a single-guide RNA (sgRNA) target site for clustered regularly interspaced short palindromic repeats-associated protein 9- (CRISPR/Cas9-) mediated mutagenesis. **(A)** Exons, introns, and untranslated regions (UTRs) are indicated by solid boxes, lines, and blank boxes, respectively. cN2-F and cN2-R are the primers for genotyping mutation, and its position is indicated by arrowheads. The mutation is identified within the target site of *OsABI5* generated through CRISPR/Cas9-mediated genome editing in rice. The PAM sequences (NGG) are boxed and the 20-nt target sequences are underlined. Mutations are shown in red letters for insertion or in “–” for deletion. **(B)** The three-dimensional structures of OsABI5 were analyzed on the SWISS-MODEL (https://www.swissmodel.expasy.org/).

Supplementary Figure 2 Effects of exogenous melatonin (MT) treatment on the concentration of **(A)** hydrogen peroxide (H_2_O_2_) and **(B)** malondialdehyde (MDA) at 15°C. The concentration of H_2_O_2_ and MDA was measured under low-temperature (LT) stress (constant 15°C for 7 days) without (LT15) or with (LT15 + MT) MT treatment in wild type (WT) and *abi5* mutants. Different letters denote significant variations between the treatments, and the average values were measured by Tukey’s honestly significant difference (HSD) test at *p* < 0.05. Data were represented as mean ± SE of six biological replicates.

Supplementary Figure 3 Effects of exogenous MT treatment on seed germination performance under LT stress. **(A)** Germinability, **(B)** germination index, **(C)** vital index, and **(D)** moisture absorption under LT stress (constant 20°C for 7 days) without [LT of constant 20°C (LT20)] or with (LT20 + MT) MT treatment. Different letters denote significant variations between the treatments, and the average values were measured by Tukey’s HSD test at *p* < 0.05. Data were represented as mean ± SE of six biological replicates.

Supplementary Figure 4 Effects of exogenous MT treatment on the acid value during seed germination under LT stress. The acid value during LT stress (constant 20°C for 7 days) without (LT20) or with MT (LT20 + MT) treatment. Different letters denote significant variations between treatments, and the average values were measured by Tukey’s HSD test at *p* < 0.05. Data were represented as mean ± SE of six biological replicates.

Supplementary Figure 5 Effects of exogenous MT treatment on endogenous abscisic acid (ABA) biosynthesis. Transcript abundance of ABA biosynthesis genes, **(A)** *OsNCED2* and **(B)** *OsAAO* under LT stress (constant 20°C for 7 days) without (LT20) or with MT (LT20 + MT) treatment in WT and *abi5* mutants. Different letters denote significant variations between the treatments, and the average values were measured by Tukey’s HSD test at *p* < 0.05. Data were represented as mean ± SE of six biological replicates.

Supplementary Figure 6 Effects of exogenous MT treatment on endogenous gibberellin (GA) biosynthesis. **(A–D)** Transcript abundance of GA of catabolic genes (*OsGA2ox1*, *OsGA2ox2*, *OsGA2ox3*, and *OsGA2ox4*) during LT stress (constant 20°C for 7 days) without (LT20) or with (LT20 + MT) MT treatment in WT and *abi5* mutants. Different letters denote significant variations between the treatments, and the average values were measured by Tukey’s HSD test at *p* < 0.05. Data were represented as mean ± SE of six biological replicates.

Supplementary Table 1 Primers used in this study.

Supplementary Table 2 Two-way ANOVA test for seed germination performance under low-temperature (LT) stress.

Supplementary Table 3 Two-way ANOVA test for seed germination-associated physiological attributes during LT stress.

Supplementary Table 4 Two-way ANOVA test for seed germination associated with enzymic activities under LT stress.

Supplementary Table 5 Two-way ANOVA test for an antioxidant system under LT.

Supplementary Table 6 Two-way ANOVA test for the biosynthesis of endogenous melatonin (MT).

Supplementary Table 7 Two-way ANOVA test for endogenous abscisic acid (ABA) biosynthesis.

Supplementary Table 8 Two-way ANOVA test for endogenous gibberellin (GA) biosynthesis.

Supplementary Table 9 Two-way ANOVA test for the *OsCAT5* expression level.
