# Supplementary material for: Melatonin Alleviates Low-Temperature Stress via ABI5-Mediated Signals During Seed Germination in Rice (Oryza sativa L.)
Source: Front Plant Sci. 2021 Sep 27;12:727596. doi: 10.3389/fpls.2021.727596 (PMC8502935; doi:10.3389/fpls.2021.727596)
Supplement: Supplementary Figure 1 — A schematic diagram of an ABA-INSENSITIVE 5 (OsABI5) and a single-guide RNA (sgRNA) target site for clustered regularly interspaced short palindromic repeats-associated protein 9- (CRISPR/Cas9-) mediated mutagenesis. (A) Exons, introns, and untranslated regions (UTRs) are indicated by solid boxes, lines, and blank boxes, respectively. cN2-F and cN2-R are the primers for genotyping mutation, and its position is indicated by arrowheads. The mutation is identified within the target site of OsABI5 generated through CRISPR/Cas9-mediated genome editing in rice. The PAM sequences (NGG) are boxed and the 20-nt target sequences are underlined. Mutations are shown in red letters for insertion or in “–” for deletion. (B) The three-dimensional structures of OsABI5 were analyzed on the SWISS-MODEL (https://www.swissmodel.expasy.org/). [file Data_Sheet_1.pdf]

Supplementary Material

1 Supplementary Figures and Tables

1.1 Supplementary Figures

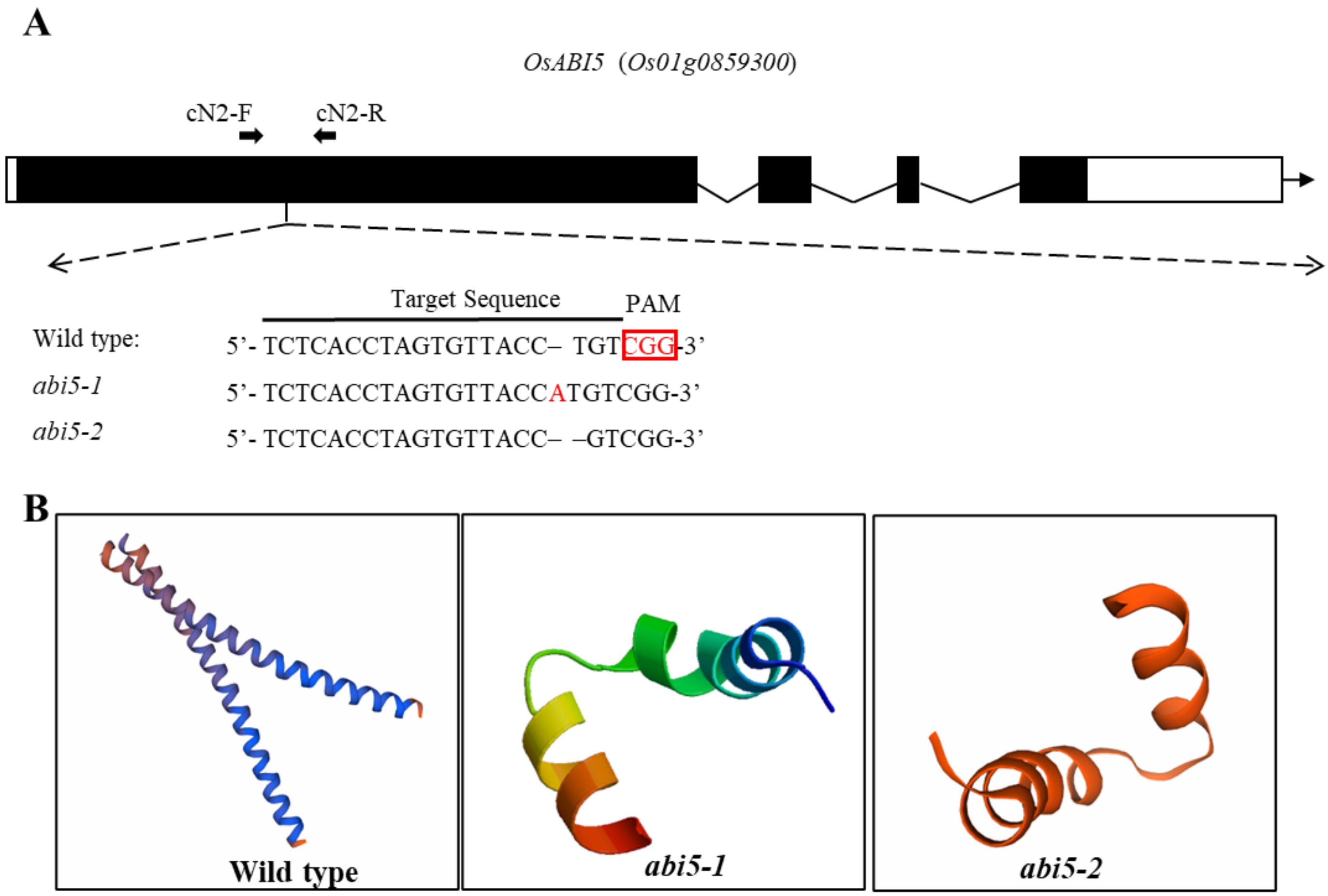

Supplementary Figure 1. Schematic diagram of *OsABI5* and sgRNA target site for CRISPR/Cas9-mediated mutagenesis. (**A**) Exons, introns and UTRs are indicated by solid boxes, lines and blank boxes, respectively. cN2-F and cN2-R are primers for genotyping mutation, and its position are indicated by arrowheads. Mutation identified within the target site of *OsABI5* generated through CRISPR/Cas9-mediated genome editing in rice. The PAM sequences (NGG) are boxed and the 20-nt target sequences are underlined. Mutations are shown in red letters for insertion or in '–' for deletion. (**B**) The 3-dimensional structures of OsABI5 were analyzed on SWISS-MODEL (https://www.swissmodel.expasy.org/).

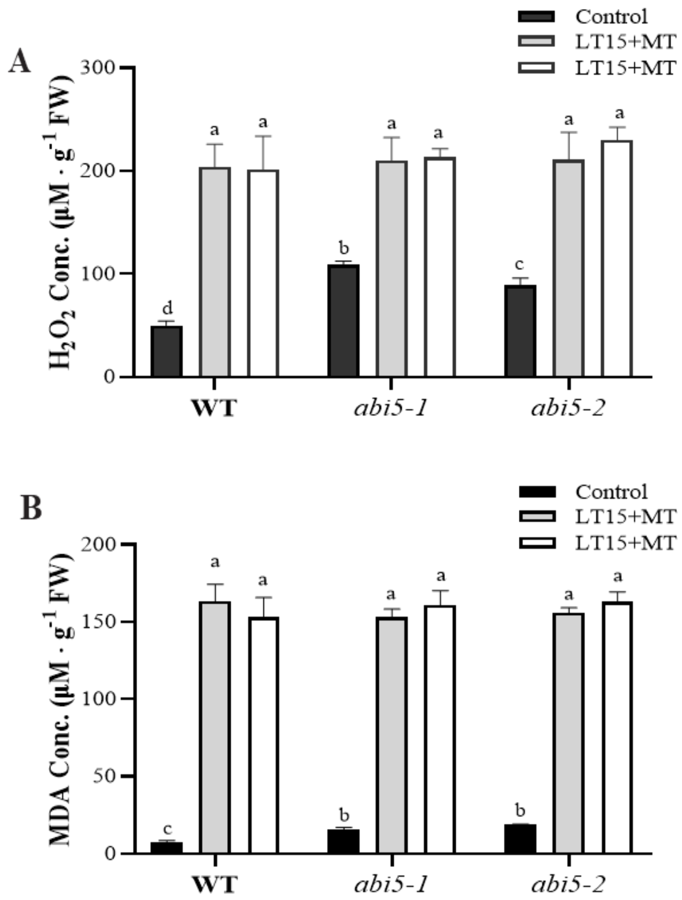

Supplementary Figure 2. Effects of exogenous melatonin treatment on the concentration of (A) hydrogen oxygen (H_2O_2) and (B) malondialdehyde (MDA) at 15℃. The concentration of (**A**) hydrogen oxygen (H_2O_2) and (**B**) malondialdehyde (MDA) during low temperature stress (LT, constant 15℃ for 7 days) without (LT15) or with (LT15+MT) melatonin (MT) treatment in wild-type (WT) and the *abi5* mutants. Different letters denote the significant variations between the treatments and the average values were measured by Tukey's Honestly Significant Difference (HSD) test at $P < 0.05$. Data represented as the mean ± standard error of six biological replicates.

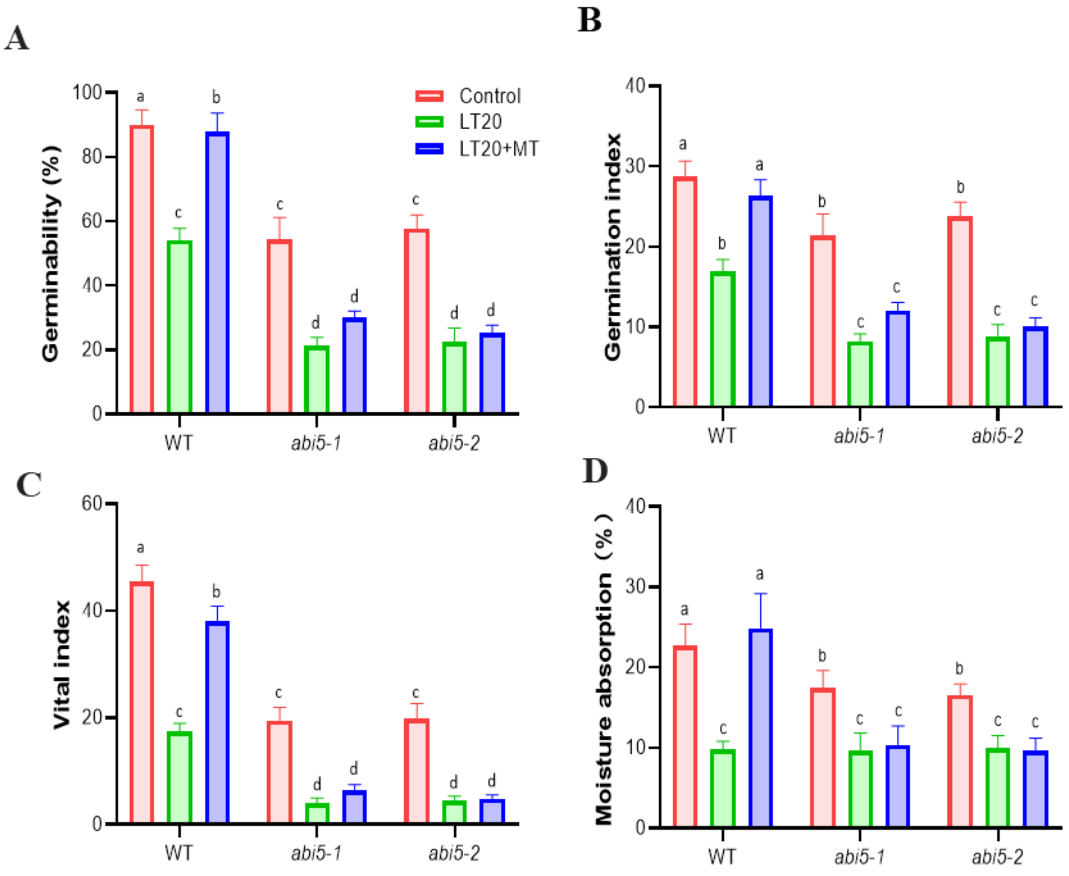

Supplementary Figure 3. Effects of exogenous melatonin treatment on seed germination performance under low temperature stress. (**A**) Germinability, (**B**) germination index, (**C**) vital index, and (**D**) moisture absorption during low temperature stress (LT, constant 20℃ for 7 days) without (LT20) or with (LT20+MT) melatonin (MT) treatment. Different letters denote the significant variations between the treatments and the average values were measured by Tukey's Honestly Significant Difference (HSD) test at $P < 0.05$. Data represented as the mean ± standard error of six biological replicates.

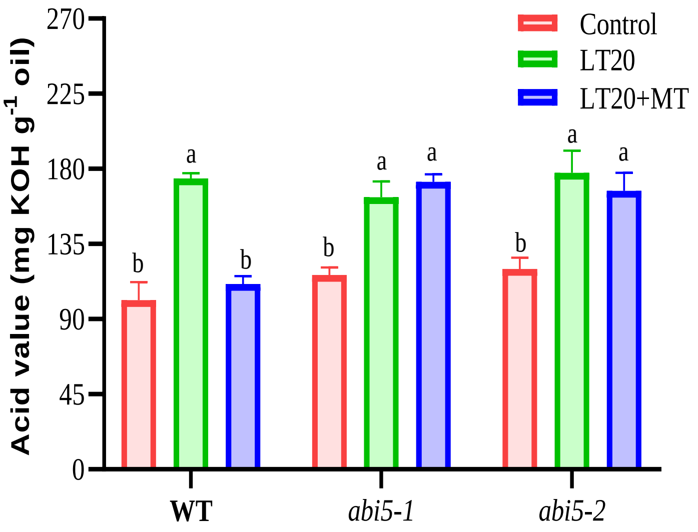

Supplementary Figure 4. Effects of exogenous melatonin treatment on acid value during seed germination under low temperature stress. Acid value during low temperature stress (LT, constant 20℃ for 7 days) without (LT20) or with (LT20+MT) melatonin (MT) treatment. Different letters denote the significant variations between the treatments and the average values were measured by Tukey's Honestly Significant Difference (HSD) test at $P < 0.05$. Data represented as the mean ± standard error of six biological replicates.

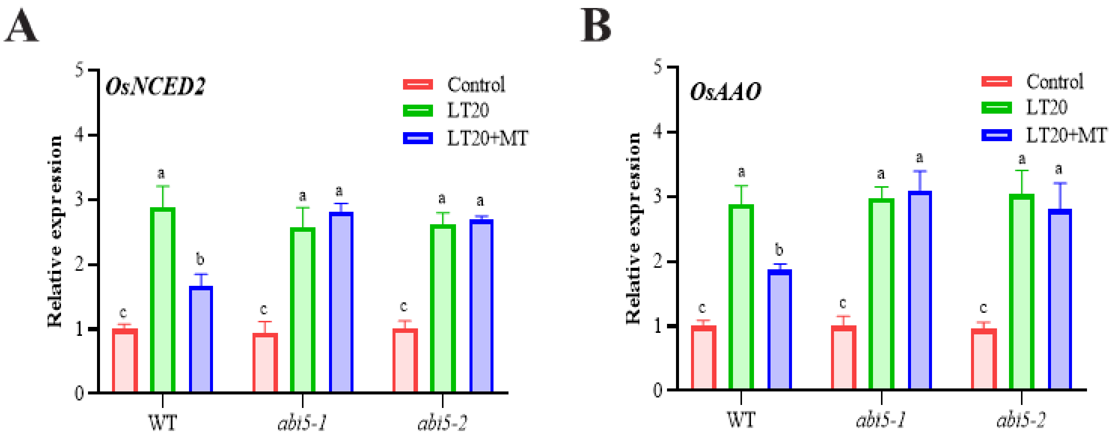

Supplementary Figure 5. Effects of exogenous melatonin treatment on the endogenous ABA biosynthesis.(**A–B**) Transcript abundance of ABA biosynthesis genes (*NCED2*, and *AAO* during low temperature stress (LT, constant 20℃ for 7 days) without (LT20) or with (LT20+MT) melatonin (MT) treatment in wild-type (WT) and the *abi5* mutants. Different letters denote the significant variations between the treatments and the average values were measured by Tukey's Honestly Significant Difference (HSD) test at $P < 0.05$. Data represented as the mean ± standard error of six biological replicates.

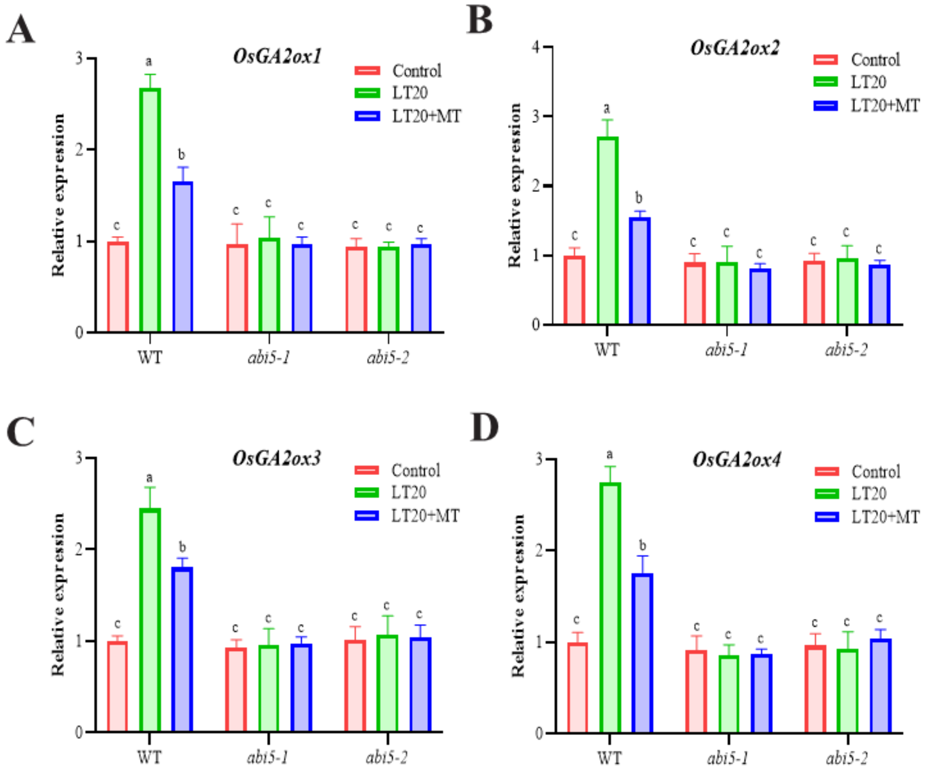

Supplementary Figure 6. Effects of exogenous melatonin treatment on the endogenous GA biosynthesis.(**A-D**) Transcript abundance of GA of catabolic genes (*GA2ox1*, *GA2ox2*, *GA2ox3* and *GA2ox4*) during low temperature stress (LT, constant 20℃ for 7 days) without (LT20) or with (LT20+MT) melatonin (MEL) treatment in wild-type (WT) and the *abi5* mutants. Different letters denote the significant variations between the treatments and the average values were measured by Tukey's Honestly Significant Difference (HSD) test at $P < 0.05$. Data represented as the mean ± standard error of six biological replicates.

1.2 Supplementary Tables

Supplementary Table 1 | Primers used in this study

Name	Forward Primer（5’→3’）	Reverse Primer（5’→3’）
L64000 pHUC411-sg2.0	GGCATCTCACCTAGTGTTACCTGT	AAACACAGGTAACACTAGGTGAGA
cN2	ATCATTTTCCTTGCCGCTACCG	ATCCAGGACTCACGACAACCAT
pCZN1-LOC	CATATGGCCAGTGAAATGAGCA	TCTAGATTACCACATACAAC
Biotin_CAT2 promoter	TGTTTGAACAACGTAGTAAATGATA	TATCATTTACTACGTTGTTCAAACA
UBQ5	CCGACTACAACATCCAGAAGGAG	AACAGGAGCCTACGCCTAAGC
TDC	GCGAGGGTGAAACCTTCCA	GCGAGCCGGTGGAGTCC
T5H	CCTCGTCCTGGACATCTTCGTC	ATGGCGAACCTCTTGATGAACAC
SNAT	GGGCTGCGGCAACTTGGTCC	AGAAAGCTCGGTCTAAAATCTGGGGTAT
ASMT	CGCCAAGGCTCCCAGTAACAAC	TGATCGTGCGCACTACTGACTCCGGC
NCED1	AGCACCAATGATACAAACCAAC	TGTGTGCACTTATACTACGTGT
NCED2	GTATGGAAACGAGGATAGTGGT	CTTCTGCAACAATGTCTAGCTC
AAO	AAGTTCTGGCTGATCTAAAGCT	CCATTATCGACACATCACACAC
ABI5	GATCATTTTCCTTGCCGCTAC	CAAGTGTCATCTCACCTAGTGT
CYP99A3	CTCAAGTTCAGCAATGGGTTC	CCAGTATAATTGCCTTGACGTG
CAT2	CCTATGCTGATACCCAAAG	CACACTGCGACCAGTAGGA
GA1	ACTAGGTTGTAAGTCACACAGG	CAATCAATGGTCCACGTGTAAA
GA2ox1	AATACCACCACTCACTCTTAGC	CATGCATACTTTGACACGTAGG
GA2ox2	GGAGTACAAGAGGACCATGTAC	CTATTCATGGTCGTCATCGTCC
GA2ox3	TCAAGCAACAACGACTTTACTG	GCTGTACATTGTGCATCTTGAA
GA2ox4	TGTCAATGATCTACTTCGGAGG	CCTTAACTTAGCTTAGGCGAGT

Supplementary Table 2 | Two-way ANOVA test for seed germination performance during low temperature stress.

**Germinability**					
Source of Variation	% of total variation	P value	P value summary	Significant?	
Interaction	5.889	<0.0001	****	Yes	
Row Factor	60.59	<0.0001	****	Yes	
Column Factor	31.12	<0.0001	****	Yes	
ANOVA table	SS	DF	MS	F (DFn, DFd)	P value
Interaction	2066	4	516.4	F (4, 45) = 27.64	P<0.0001
Row Factor	21254	2	10627	F (2, 45) = 568.7	P<0.0001
Column Factor	10918	2	5459	F (2, 45) = 292.1	P<0.0001
Residual	840.9	45	18.69		
**Germination index**					
Source of Variation	% of total variation	P value	P value summary	Significant?	
Interaction	6.591	<0.0001	****	Yes	
Row Factor	37.69	<0.0001	****	Yes	
Column Factor	51.77	<0.0001	****	Yes	
ANOVA table	SS	DF	MS	F (DFn, DFd)	P value
Interaction	208.6	4	52.16	F (4, 45) = 18.75	P<0.0001
Row Factor	1193	2	596.6	F (2, 45) = 214.5	P<0.0001
Column Factor	1639	2	819.4	F (2, 45) = 294.6	P<0.0001
Residual	125.2	45	2.782		
**Vital index**					
Source of Variation	% of total variation	P value	P value summary	Significant?	
Interaction	6.879	<0.0001	****	Yes	
Row Factor	60.28	<0.0001	****	Yes	
Column Factor	31.22	<0.0001	****	Yes	
ANOVA table	SS	DF	MS	F (DFn, DFd)	P value
Interaction	776.3	4	194.1	F (4, 45) = 47.95	P<0.0001
Row Factor	6803	2	3401	F (2, 45) = 840.3	P<0.0001
Column Factor	3523	2	1762	F (2, 45) = 435.2	P<0.0001
Residual	182.1	45	4.048		
**Moisture absorption**					
Source of Variation	% of total variation	P value	P value summary	Significant?	
Interaction	22.2	<0.0001	****	Yes	
Row Factor	28.44	<0.0001	****	Yes	
Column Factor	37.3	<0.0001	****	Yes	
ANOVA table	SS	DF	MS	F (DFn, DFd)	P value
Interaction	449	4	112.3	F (4, 45) = 20.72	P<0.0001
Row Factor	575.1	2	287.6	F (2, 45) = 53.07	P<0.0001
Column Factor	754.2	2	377.1	F (2, 45) = 69.59	P<0.0001

Residual	243.9	45	5.419		
**Germination percentage**					
Source of Variation	% of total variation	P value	P value summary	Significant?	
Interaction	3.994	<0.0001	****	Yes	
Row Factor	66.31	<0.0001	****	Yes	
Column Factor	28.07	<0.0001	****	Yes	
ANOVA table	SS	DF	MS	F (DFn, DFd)	P value
Interaction	1575	4	393.8	F (4, 45) = 27.62	P<0.0001
Row Factor	26153	2	13076	F (2, 45) = 917.1	P<0.0001
Column Factor	11069	2	5535	F (2, 45) = 388.1	P<0.0001
**Healthy seedling**					
Source of Variation	% of total variation	P value	P value summary	Significant?	
Interaction	9.588	<0.0001	****	Yes	
Row Factor	54.84	<0.0001	****	Yes	
Column Factor	34.35	<0.0001	****	Yes	
ANOVA table	SS	DF	MS	F (DFn, DFd)	P value
Interaction	4408	4	1102	F (4, 45) = 88.24	P<0.0001
Row Factor	25209	2	12605	F (2, 45) = 1009	P<0.0001
Column Factor	15791	2	7895	F (2, 45) = 632.3	P<0.0001
Residual	561.9	45	12.49		
**Fig.S3A H_2O_2 CONTENT**					
Source of Variation	% of total variation	P value	P value summary	Significant?	
Interaction	19.25	<0.0001	****	Yes	
Row Factor	22.79	<0.0001	****	Yes	
Column Factor	54.25	<0.0001	****	Yes	
ANOVA table	SS	DF	MS	F (DFn, DFd)	P value
Interaction	52535	4	13134	F (4, 45) = 58.50	P<0.0001
Row Factor	62202	2	31101	F (2, 45) = 138.5	P<0.0001
Column Factor	148061	2	74030	F (2, 45) = 329.7	P<0.0001
Residual	10103	45	224.5		
**Fig.S3B MDA CONTENT**					
Source of Variation	% of total variation	P value	P value summary	Significant?	
Interaction	21.98	<0.0001	****	Yes	
Row Factor	10.97	<0.0001	****	Yes	
Column Factor	66.54	<0.0001	****	Yes	
ANOVA table	SS	DF	MS	F (DFn, DFd)	P value
Interaction	63770	4	15943	F (4, 45) = 494.1	P<0.0001
Row Factor	31836	2	15918	F (2, 45) = 493.3	P<0.0001
Column Factor	193045	2	96523	F (2, 45) = 2991	P<0.0001
Residual	1452	45	32.27		

Supplementary Table 3 | Two-way ANOVA test for seed germination associated physiological attributes during low temperature stress

**Fig. 3A Physical efficiency**					
Source of Variation	% of total variation	P value	P value summary	Significant?	
Interaction	12.1	<0.0001	****	Yes	
Row Factor	69.11	<0.0001	****	Yes	
Column Factor	17.13	<0.0001	****	Yes	
ANOVA table	SS	DF	MS	F (DFn, DFd)	P value
Interaction	1561	4	390.2	F (4, 45) = 82.07	P<0.0001
Row Factor	8919	2	4459	F (2, 45) = 937.8	P<0.0001
Column Factor	2211	2	1106	F (2, 45) = 232.5	P<0.0001
Residual	214	45	4.755		
**Fig. 3B Soluble sugars**					
Source of Variation	% of total variation	P value	P value summary	Significant?	
Interaction	8.146	<0.0001	****	Yes	
Row Factor	47.92	<0.0001	****	Yes	
Column Factor	38.07	<0.0001	****	Yes	
ANOVA table	SS	DF	MS	F (DFn, DFd)	P value
Interaction	1648	4	412.1	F (4, 45) = 15.62	P<0.0001
Row Factor	9696	2	4848	F (2, 45) = 183.8	P<0.0001
Column Factor	7703	2	3852	F (2, 45) = 146.0	P<0.0001
Residual	1187	45	26.37		
**Fig. 3C Soluble protein**					
Source of Variation	% of total variation	P value	P value summary	Significant?	
Interaction	13.15	<0.0001	****	Yes	
Row Factor	48.61	<0.0001	****	Yes	
Column Factor	36.97	<0.0001	****	Yes	
ANOVA table	SS	DF	MS	F (DFn, DFd)	P value
Interaction	2.733	4	0.6833	F (4, 45) = 116.4	P<0.0001
Row Factor	10.1	2	5.052	F (2, 45) = 860.5	P<0.0001
Column Factor	7.683	2	3.841	F (2, 45) = 654.3	P<0.0001
Residual	0.2642	45	0.005871		
**Fig. 3D Phospholipid**					
Source of Variation	% of total variation	P value	P value summary	Significant?	
Interaction	13.27	<0.0001	****	Yes	
Row Factor	54.63	<0.0001	****	Yes	
Column Factor	31.77	<0.0001	****	Yes	
ANOVA table	SS	DF	MS	F (DFn, DFd)	P value
Interaction	0.02635	4	0.006587	F (4, 45) = 468.8	P<0.0001
Row Factor	0.1084	2	0.05422	F (2, 45) = 3859	P<0.0001

Column Factor	0.06307	2	0.03153	F (2, 45) = 2245	P<0.0001
Residual	0.0006322	45	0.00001405		
**Fig. 3E Respiratory rates**					
Source of Variation	% of total variation	P value	P value summary	Significant?	
Interaction	45.79	<0.0001	****	Yes	
Row Factor	30.44	<0.0001	****	Yes	
Column Factor	22.95	<0.0001	****	Yes	
ANOVA table	SS	DF	MS	F (DFn, DFd)	P value
Interaction	86.73	4	21.68	F (4, 45) = 625.7	P<0.0001
Row Factor	57.64	2	28.82	F (2, 45) = 831.7	P<0.0001
Column Factor	43.46	2	21.73	F (2, 45) = 627.1	P<0.0001
Residual	1.56	45	0.03466		
**Fig.3F Membrane permeability**					
Source of Variation	% of total variation	P value	P value summary	Significant?	
Interaction	12.92	<0.0001	****	Yes	
Row Factor	23.34	<0.0001	****	Yes	
Column Factor	61.93	<0.0001	****	Yes	
ANOVA table	SS	DF	MS	F (DFn, DFd)	P value
Interaction	0.2783	4	0.06959	F (4, 45) = 80.05	P<0.0001
Row Factor	0.5029	2	0.2514	F (2, 45) = 289.2	P<0.0001
Column Factor	1.334	2	0.6672	F (2, 45) = 767.6	P<0.0001
Residual	0.03912	45	0.0008693		
**Fig. S4 Acid value**					
Source of Variation	% of total variation	P value	P value summary	Significant?	
Interaction	17.06	<0.0001	****	Yes	
Row Factor	13.72	<0.0001	****	Yes	
Column Factor	63.12	<0.0001	****	Yes	
ANOVA table	SS	DF	MS	F (DFn, DFd)	P value
Interaction	8690	4	2173	F (4, 45) = 31.45	P<0.0001
Row Factor	6987	2	3494	F (2, 45) = 50.58	P<0.0001
Column Factor	32148	2	16074	F (2, 45) = 232.7	P<0.0001
Residual	3108	45	69.08		

Supplementary Table 4 | Two-way ANOVA test for seed germination associated with enzymic activities during low temperature stress

**Fig. 4A α-amylose**					
Source of Variation	% of total variation	P value	P value summary	Significant?	
Interaction	15.69	<0.0001	****	Yes	
Row Factor	12.11	<0.0001	****	Yes	
Column Factor	68.03	<0.0001	****	Yes	
ANOVA table	SS	DF	MS	F (DFn, DFd)	P value
Interaction	222.3	4	55.56	F (4, 45) = 42.32	P<0.0001
Row Factor	171.5	2	85.75	F (2, 45) = 65.31	P<0.0001
Column Factor	963.5	2	481.8	F (2, 45) = 366.9	P<0.0001
Residual	59.09	45	1.313		
**Fig. 4B Acid phosphatase**					
Source of Variation	% of total variation	P value	P value summary	Significant?	
Interaction	13.97	<0.0001	****	Yes	
Row Factor	20.64	<0.0001	****	Yes	
Column Factor	60.87	<0.0001	****	Yes	
ANOVA table	SS	DF	MS	F (DFn, DFd)	P value
Interaction	197.4	4	49.35	F (4, 45) = 34.75	P<0.0001
Row Factor	291.7	2	145.9	F (2, 45) = 102.7	P<0.0001
Column Factor	860.1	2	430	F (2, 45) = 302.8	P<0.0001
Residual	63.91	45	1.42		
**Fig.4C SDH**					
Source of Variation	% of total variation	P value	P value summary	Significant?	
Interaction	15.31	<0.0001	****	Yes	
Row Factor	75.07	<0.0001	****	Yes	
Column Factor	8.084	<0.0001	****	Yes	
ANOVA table	SS	DF	MS	F (DFn, DFd)	P value
Interaction	2.933	4	0.7332	F (4, 45) = 112.2	P<0.0001
Row Factor	14.38	2	7.188	F (2, 45) = 1100	P<0.0001
Column Factor	1.548	2	0.7741	F (2, 45) = 118.4	P<0.0001
Residual	0.2942	45	0.006537		
**Fig.4D CCO**					

Source of Variation	% of total variation	P value	P value summary	Significant?	
Interaction	18.96	<0.0001	****	Yes	
Row Factor	69.81	<0.0001	****	Yes	
Column Factor	9.988	<0.0001	****	Yes	
ANOVA table	SS	DF	MS	F (DFn, DFd)	P value
Interaction	418.4	4	104.6	F (4, 45) = 171.5	P<0.0001
Row Factor	1540	2	770.1	F (2, 45) = 1263	P<0.0001
Column Factor	220.4	2	110.2	F (2, 45) = 180.6	P<0.0001
Residual	27.45	45	0.61		

Supplementary Table 5 | Two-way ANOVA test for the antioxidant system under low temperature

**Fig.5A_H_2O_2**					
Source of Variation	% of total variation	P value	P value summary	Significant?	
Interaction	5.569	<0.0001	****	Yes	
Row Factor	70.3	<0.0001	****	Yes	
Column Factor	22.19	<0.0001	****	Yes	
ANOVA table	SS	DF	MS	F (DFn, DFd)	P value
Interaction	2404	4	601.1	F (4, 45) = 32.26	P<0.0001
Row Factor	30353	2	15176	F (2, 45) = 814.5	P<0.0001
Column Factor	9578	2	4789	F (2, 45) = 257.0	P<0.0001
Residual	838.5	45	18.63		
**Fig.5B MDA**					
Source of Variation	% of total variation	P value	P value summary	Significant?	
Interaction	8.521	<0.0001	****	Yes	
Row Factor	64.4	<0.0001	****	Yes	
Column Factor	21.89	<0.0001	****	Yes	
ANOVA table	SS	DF	MS	F (DFn, DFd)	P value
Interaction	229.3	4	57.32	F (4, 45) = 18.48	P<0.0001
Row Factor	1733	2	866.4	F (2, 45) = 279.3	P<0.0001
Column Factor	589	2	294.5	F (2, 45) = 94.94	P<0.0001
Residual	139.6	45	3.102		
**Fig.5C Proline**					
Source of Variation	% of total variation	P value	P value summary	Significant?	
Interaction	9.366	<0.0001	****	Yes	
Row Factor	76.77	<0.0001	****	Yes	
Column Factor	11.65	<0.0001	****	Yes	
ANOVA table	SS	DF	MS	F (DFn, DFd)	P value
Interaction	36.63	4	9.156	F (4, 45) = 47.62	P<0.0001
Row Factor	300.2	2	150.1	F (2, 45) = 780.8	P<0.0001
Column Factor	45.55	2	22.78	F (2, 45) = 118.5	P<0.0001
Residual	8.652	45	0.1923		
**Fig.5D CAT**					
Source of Variation	% of total variation	P value	P value summary	Significant?	
Interaction	26.84	<0.0001	****	Yes	
Row Factor	57.64	<0.0001	****	Yes	
Column Factor	14.22	<0.0001	****	Yes	
ANOVA table	SS	DF	MS	F (DFn, DFd)	P value
Interaction	17474	4	4368	F (4, 45) = 232.2	P<0.0001
Row Factor	37527	2	18763	F (2, 45) = 997.5	P<0.0001

Column Factor	9257	2	4629	F (2, 45) = 246.1	P<0.0001
Residual	846.5	45	18.81		
**Fig.5E SOD**					
Source of Variation	% of total variation	P value	P value summary	Significant?	
Interaction	27.81	<0.0001	****	Yes	
Row Factor	52.04	<0.0001	****	Yes	
Column Factor	14.67	<0.0001	****	Yes	
ANOVA table	SS	DF	MS	F (DFn, DFd)	P value
Interaction	68819	4	17205	F (4, 45) = 57.16	P<0.0001
Row Factor	128789	2	64395	F (2, 45) = 213.9	P<0.0001
Column Factor	36313	2	18156	F (2, 45) = 60.32	P<0.0001
Residual	13545	45	301		
**Fig.5F POD**					
Source of Variation	% of total variation	P value	P value summary	Significant?	
Interaction	37.24	<0.0001	****	Yes	
Row Factor	42.9	<0.0001	****	Yes	
Column Factor	18.19	<0.0001	****	Yes	
ANOVA table	SS	DF	MS	F (DFn, DFd)	P value
Interaction	50625	4	12656	F (4, 45) = 249.7	P<0.0001
Row Factor	58327	2	29164	F (2, 45) = 575.5	P<0.0001
Column Factor	24725	2	12363	F (2, 45) = 243.9	P<0.0001
Residual	2281	45	50.68		

Supplementary Table 6 | Two-way ANOVA test for the biosynthesis of endogenous melatonin

**Fig.6A Melatonin Content**					
Source of Variation	% of total variation	P value	P value summary	Significant?	
Interaction	30	<0.0001	****	Yes	
Row Factor	38.69	<0.0001	****	Yes	
Column Factor	13.73	<0.0001	****	Yes	
ANOVA table	SS	DF	MS	F (DFn, DFd)	P value
Interaction	9.238	4	2.31	F (4, 45) = 19.18	P<0.0001
Row Factor	11.91	2	5.957	F (2, 45) = 49.48	P<0.0001
Column Factor	4.228	2	2.114	F (2, 45) = 17.56	P<0.0001
Residual	5.418	45	0.1204		
**Fig.6B *OsTDC* expression level**					
Source of Variation	% of total variation	P value	P value summary	Significant?	
Interaction	31.5	<0.0001	****	Yes	
Row Factor	50.3	<0.0001	****	Yes	
Column Factor	16.96	<0.0001	****	Yes	
ANOVA table	SS	DF	MS	F (DFn, DFd)	P value
Interaction	23.83	4	5.959	F (4, 45) = 288.1	P<0.0001
Row Factor	38.06	2	19.03	F (2, 45) = 920.1	P<0.0001
Column Factor	12.84	2	6.418	F (2, 45) = 310.3	P<0.0001
Residual	0.9307	45	0.02068		
**Fig.6C *OsT5H* expression level**					
Source of Variation	% of total variation	P value	P value summary	Significant?	
Interaction	32.44	<0.0001	****	Yes	
Row Factor	47.73	<0.0001	****	Yes	
Column Factor	16.41	<0.0001	****	Yes	
ANOVA table	SS	DF	MS	F (DFn, DFd)	P value
Interaction	19.17	4	4.792	F (4, 45) = 106.6	P<0.0001
Row Factor	28.2	2	14.1	F (2, 45) = 313.7	P<0.0001
Column Factor	9.693	2	4.847	F (2, 45) = 107.8	P<0.0001
Residual	2.022	45	0.04494		
**Fig.6D *OsSNAT* expression level**					
Source of Variation	% of total variation	P value	P value summary	Significant?	
Interaction	31.58	<0.0001	****	Yes	
Row Factor	49.65	<0.0001	****	Yes	

Column Factor	16.71	<0.0001	****	Yes	
ANOVA table	SS	DF	MS	F (DFn, DFd)	P value
Interaction	25.98	4	6.495	F (4, 45) = 172.3	P<0.0001
Row Factor	40.85	2	20.43	F (2, 45) = 541.8	P<0.0001
Column Factor	13.75	2	6.875	F (2, 45) = 182.4	P<0.0001
Residual	1.697	45	0.0377		
**Fig.6E *OsASMT* expression level**					
Source of Variation	% of total variation	P value	P value summary	Significant?	
Interaction	34.86	<0.0001	****	Yes	
Row Factor	43.4	<0.0001	****	Yes	
Column Factor	17.82	<0.0001	****	Yes	
ANOVA table	SS	DF	MS	F (DFn, DFd)	P value
Interaction	21.39	4	5.348	F (4, 45) = 100.2	P<0.0001
Row Factor	26.64	2	13.32	F (2, 45) = 249.6	P<0.0001
Column Factor	10.94	2	5.469	F (2, 45) = 102.5	P<0.0001
Residual	2.402	45	0.05337		

Supplementary Table 7 | Two-way ANOVA test for endogenous ABA biosynthesis

**Fig.7A ABA Content**					
Source of Variation	% of total variation	P value	P value summary	Significant?	
Interaction	10.33	<0.0001	****	Yes	
Row Factor	5.912	<0.0001	****	Yes	
Column Factor	81.12	<0.0001	****	Yes	
ANOVA table	SS	DF	MS	F (DFn, DFd)	P value
Interaction	3.581	4	0.8954	F (4, 45) = 44.16	P<0.0001
Row Factor	2.049	2	1.024	F (2, 45) = 50.53	P<0.0001
Column Factor	28.11	2	14.06	F (2, 45) = 693.3	P<0.0001
Residual	0.9124	45	0.02027		
**Fig.7B *OsNCED1* expression level**					
Source of Variation	% of total variation	P value	P value summary	Significant?	
Interaction	8.694	<0.0001	****	Yes	
Row Factor	3.475	<0.0001	****	Yes	
Column Factor	81.05	<0.0001	****	Yes	
ANOVA table	SS	DF	MS	F (DFn, DFd)	P value
Interaction	2.368	4	0.5921	F (4, 45) = 14.43	P<0.0001
Row Factor	0.9468	2	0.4734	F (2, 45) = 11.54	P<0.0001
Column Factor	22.08	2	11.04	F (2, 45) = 269.1	P<0.0001
Residual	1.846	45	0.04102		
**Fig.7C *OsABI5* expression level**					
Source of Variation	% of total variation	P value	P value summary	Significant?	
Interaction	22.85	<0.0001	****	Yes	
Row Factor	65.07	<0.0001	****	Yes	
Column Factor	11.65	<0.0001	****	Yes	
ANOVA table	SS	DF	MS	F (DFn, DFd)	P value
Interaction	39.46	4	9.866	F (4, 45) = 602.0	P<0.0001
Row Factor	112.4	2	56.18	F (2, 45) = 3428	P<0.0001
Column Factor	20.11	2	10.06	F (2, 45) = 613.7	P<0.0001
Residual	0.7375	45	0.01639		
**Fig.7D *OsCYP99A3* expression level**					
Source of Variation	% of total variation	P value	P value summary	Significant?	
Interaction	22.55	<0.0001	****	Yes	
Row Factor	61.08	<0.0001	****	Yes	
Column Factor	14.47	<0.0001	****	Yes	
ANOVA table	SS	DF	MS	F (DFn, DFd)	P value

Interaction	1.606	4	0.4015	F (4, 45) = 133.6	P<0.0001
Row Factor	4.35	2	2.175	F (2, 45) = 723.8	P<0.0001
Column Factor	1.03	2	0.5151	F (2, 45) = 171.4	P<0.0001
Residual	0.1352	45	0.003005		
**Fig.S5A *OsNCED2* expression level**					
Source of Variation	% of total variation	P value	P value summary	Significant?	
Interaction	11.76	<0.0001	****	Yes	
Row Factor	2.279	0.0001	***	Yes	
Column Factor	81.32	<0.0001	****	Yes	
ANOVA table	SS	DF	MS	F (DFn, DFd)	P value
Interaction	4.305	4	1.076	F (4, 45) = 28.52	P<0.0001
Row Factor	0.8345	2	0.4173	F (2, 45) = 11.06	P=0.0001
Column Factor	29.78	2	14.89	F (2, 45) = 394.5	P<0.0001
Residual	1.698	45	0.03773		
**Fig.S5B *OsAAO* expression level**					
Source of Variation	% of total variation	P value	P value summary	Significant?	
Interaction	6.583	<0.0001	****	Yes	
Row Factor	4.079	<0.0001	****	Yes	
Column Factor	83.52	<0.0001	****	Yes	
ANOVA table	SS	DF	MS	F (DFn, DFd)	P value
Interaction	3.124	4	0.7811	F (4, 45) = 12.72	P<0.0001
Row Factor	1.936	2	0.968	F (2, 45) = 15.76	P<0.0001
Column Factor	39.64	2	19.82	F (2, 45) = 322.8	P<0.0001
Residual	2.763	45	0.0614		

Supplementary Table 8 | Two-way ANOVA test for the endogenous GA biosynthesis

**Fig.7E GA content**					
Source of Variation	% of total variation	P value	P value summary	Significant?	
Interaction	7.851	<0.0001	****	Yes	
Row Factor	83.62	<0.0001	****	Yes	
Column Factor	4.574	<0.0001	****	Yes	
ANOVA table	SS	DF	MS	F (DFn, DFd)	P value
Interaction	2.755	4	0.6887	F (4, 45) = 22.32	P<0.0001
Row Factor	29.34	2	14.67	F (2, 45) = 475.5	P<0.0001
Column Factor	1.605	2	0.8025	F (2, 45) = 26.01	P<0.0001
Residual	1.388	45	0.03085		
**Fig.7F *OsGA1* expression level**					
Source of Variation	% of total variation	P value	P value summary	Significant?	
Interaction	27.68	<0.0001	****	Yes	
Row Factor	53.66	<0.0001	****	Yes	
Column Factor	16.03	<0.0001	****	Yes	
ANOVA table	SS	DF	MS	F (DFn, DFd)	P value
Interaction	8.92	4	2.23	F (4, 45) = 118.4	P<0.0001
Row Factor	17.29	2	8.645	F (2, 45) = 459.1	P<0.0001
Column Factor	5.165	2	2.583	F (2, 45) = 137.2	P<0.0001
Residual	0.8474	45	0.01883		
**Fig.S6A *OsGA2ox1* expression level**					
Source of Variation	% of total variation	P value	P value summary	Significant?	
Interaction	31.92	<0.0001	****	Yes	
Row Factor	45.15	<0.0001	****	Yes	
Column Factor	17.99	<0.0001	****	Yes	
ANOVA table	SS	DF	MS	F (DFn, DFd)	P value
Interaction	5.484	4	1.371	F (4, 45) = 72.68	P<0.0001
Row Factor	7.756	2	3.878	F (2, 45) = 205.6	P<0.0001
Column Factor	3.091	2	1.546	F (2, 45) = 81.94	P<0.0001
Residual	0.8488	45	0.01886		
**Fig.S6B *OsGA2ox2* expression level**					
Source of Variation	% of total variation	P value	P value summary	Significant?	
Interaction	30.82	<0.0001	****	Yes	
Row Factor	46.61	<0.0001	****	Yes	
Column Factor	17.41	<0.0001	****	Yes	

ANOVA table	SS	DF	MS	F (DFn, DFd)	P value
Interaction	5.884	4	1.471	F (4, 45) = 67.21	P<0.0001
Row Factor	8.899	2	4.45	F (2, 45) = 203.3	P<0.0001
Column Factor	3.324	2	1.662	F (2, 45) = 75.94	P<0.0001
Residual	0.985	45	0.02189		
**Fig.S6C *OsGA2ox3* expression level**					
Source of Variation	% of total variation	P value	P value summary	Significant?	
Interaction	28.12	<0.0001	****	Yes	
Row Factor	48.52	<0.0001	****	Yes	
Column Factor	16.74	<0.0001	****	Yes	
ANOVA table	SS	DF	MS	F (DFn, DFd)	P value
Interaction	3.969	4	0.9923	F (4, 45) = 47.72	P<0.0001
Row Factor	6.848	2	3.424	F (2, 45) = 164.7	P<0.0001
Column Factor	2.362	2	1.181	F (2, 45) = 56.80	P<0.0001
Residual	0.9357	45	0.02079		
**Fig.S6D *OsGA2ox4* expression level**					
Source of Variation	% of total variation	P value	P value summary	Significant?	
Interaction	32.14	<0.0001	****	Yes	
Row Factor	49.7	<0.0001	****	Yes	
Column Factor	13.73	<0.0001	****	Yes	
ANOVA table	SS	DF	MS	F (DFn, DFd)	P value
Interaction	6.424	4	1.606	F (4, 45) = 81.51	P<0.0001
Row Factor	9.934	2	4.967	F (2, 45) = 252.1	P<0.0001
Column Factor	2.744	2	1.372	F (2, 45) = 69.62	P<0.0001
Residual	0.8867	45	0.0197		

Supplementary Table 9 | Two-way ANOVA test for the *OsCAT5* expression level

**Fig.8A *OsCAT2* expression level**					
Source of Variation	% of total variation	P value	P value summary	Significant?	
Interaction	23.06	<0.0001	****	Yes	
Row Factor	63.37	<0.0001	****	Yes	
Column Factor	11.15	<0.0001	****	Yes	
ANOVA table	SS	DF	MS	F (DFn, DFd)	P value
Interaction	48.74	4	12.18	F (4, 45) = 107.6	P<0.0001
Row Factor	133.9	2	66.96	F (2, 45) = 591.5	P<0.0001
Column Factor	23.57	2	11.78	F (2, 45) = 104.1	P<0.0001
Residual	5.094	45	0.1132		
